# Chromosomal instability in enterohaemorrhagic *Escherichia coli* O157:H7: impact on adherence, tellurite resistance and colony phenotype

**DOI:** 10.1111/j.1365-2958.2010.07499.x

**Published:** 2011-01-04

**Authors:** Martina Bielaszewska, Barbara Middendorf, Phillip I Tarr, Wenlan Zhang, Rita Prager, Thomas Aldick, Ulrich Dobrindt, Helge Karch, Alexander Mellmann

**Affiliations:** 1 Institute of Hygiene and the National Consulting Laboratory on Haemolytic Uraemic Syndrome, University of MünsterRobert Koch Str. 41, 48149 Münster, Germany; 2 Interdisciplinary Centre of Clinical Research (IZKF) Münster, University of MünsterDomagkstr. 3, 48149 Münster, Germany; 3 Division of Paediatric Gastroenterology and Nutrition, Edward Mallinckrodt Department of Paediatrics, Washington University School of Medicine and St. Louis Children's Hospital660 South Euclid Avenue, St. Louis, MO 63110, USA; 4 National Reference Centre for Salmonella and Other Bacterial Enteric Pathogens, Robert Koch InstituteBranch Wernigerode, Burgstr. 37, 38855 Wernigerode, Germany; 5 Institute for Molecular Infection Biology, University of WürzburgJosef-Schneider-Str. 2, 97080 Würzburg, Germany

## Abstract

Tellurite (Tel) resistant enterohaemorrhagic *Escherichia coli* (EHEC) O157:H7 is a global pathogen. In strain EDL933 Tel resistance (Tel^R^) is encoded by duplicate *ter* cluster in O islands (OI) 43 and 48, which also harbour *iha*, encoding the adhesin and siderophore receptor Iha. We identified five EHEC O157:H7 strains that differentiate into large (L) colonies and small (S) colonies with high and low Tel minimal inhibitory concentrations (MICs) respectively. S colonies (Tel-MICs ≤ 4 µg ml^−1^) sustained large internal deletions within the Tel^R^ OIs via homologous recombination between IS elements and lost *ter* and *iha*. Moreover, complete excision of the islands occurred by site-specific recombination between flanking direct repeats. Complete excision of OI 43 and OI 48 occurred in 1.81 × 10^−3^ and 1.97 × 10^−4^ cells in culture, respectively; internal deletion of OI 48 was more frequent (9.7 × 10^−1^ cells). Under iron limitation that promotes *iha* transcription, *iha*-negative derivatives adhered less well to human intestinal epithelial cells and grew slower than did their *iha*-positive counterparts. Experiments utilizing *iha* deletion and complementation mutants identified Iha as the major factor responsible for these phenotypic differences. Spontaneous deletions affecting Tel^R^ OIs contribute to EHEC O157 genome plasticity and might impair virulence and/or fitness.

## Introduction

Enterohaemorrhagic *Escherichia coli* (EHEC) O157:H7 is the predominant Shiga toxin (Stx)-producing pathogen of humans (Karch *et al*., 2005; Tarr *et al*., 2005). EHEC O157:H7 resists the highly toxic tellurium oxyanion, tellurite (TeO_3_^2−^; Tel), and therefore grows in concentrations of Tel that inhibit most other *E. coli* (Zadik *et al*., 1993; Taylor *et al*., 2002; Bielaszewska *et al*., 2005; Orth *et al*., 2007). This characteristic, together with its inability to ferment sorbitol, has been exploited in selective strategies to isolate EHEC O157:H7 from faeces, food and the environment using Tel-containing media, such as cefixime-Tel sorbitol MacConkey agar (CT-SMAC) (Onoue *et al*., 1999; Van Duynhoven *et al*., 2002).

EHEC O157:H7 Tel resistance (Tel^R^) is encoded by the chromosomal *terZABCDEF* cluster (Taylor *et al*., 2002; Bielaszewska *et al*., 2005), which is highly homologous to the *ter* cluster on plasmid R478 in *Serratia marcescens* (Whelan *et al*., 1995; Taylor *et al*., 2002). EHEC O157:H7 strain EDL933 contains two identical copies of the *ter* gene cluster within identical O islands (OI) 43 and 48, which are integrated in tRNA genes *serW* and *serX*, respectively (Perna *et al*., 2001). In contrast, O157 Sakai outbreak strain RIMD 0509952 harbours only a single *ter* cluster-containing island (SpLE1) integrated in *serX* (Hayashi *et al*., 2001). This OI is also termed the Tel^R^ and adherence-conferring island, based on its first description in EHEC O157:H7 strain 86–24, because it also encodes the iron-regulated gene A (IrgA) homologue adhesin (Iha) (Tarr *et al*., 2000).

Tel^R^ is a common, but not obligatory, feature of EHEC O157:H7. Tel-susceptible *E. coli* O157:H7 strains have been isolated in North America (Taylor *et al*., 2002) and Europe (Bielaszewska *et al*., 2005). Tel susceptibility (Tel^S^) is related to lack of *ter* genes (Taylor *et al*., 2002; Bielaszewska *et al*., 2005), but mechanisms underlying the *ter* absence (i.e. loss of *ter* genes during infection or after shedding, or primary non-possession) are unknown. Here, we report the frequency of and mechanisms for loss of the *ter* gene cluster in EHEC O157:H7 clinical isolates during laboratory passage. We characterized phenotypes associated with loss of the *ter* cluster and adjacent genes, in particular *iha*. Our data suggest that full or partial deletions of the Tel^R^ island(s) diminish the virulence and/or fitness of this pathogen.

## Results

### Two different colony phenotypes in *E. coli* O157:H7 differ by susceptibility to tellurite

**Fig. 1:**
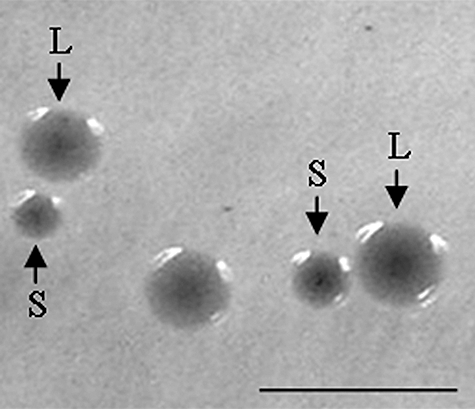
Different colony phenotypes of *E. coli* O157:H7 strains on sorbitol MacConkey agar. L, large colonies; S, small colonies. Bar represents 5 mm.

We identified five *E. coli* O157:H7 clinical isolates that produced two different colony morphologies during subculture on SMAC agar: typical large (L) and atypical small (S) colonies (Fig. 1). Both variants were confirmed to be *E. coli* O157:H7 by O:H serotyping and presence of *rfbE*_O157_ and *fliC*_H7_ (Table 1). Of the eight phenotypes we initially tested (Table 1), the L and S colonies (hereafter designated L and S strains, respectively, if they are derived from a single parent isolate) differed only in their susceptibilities to Tel. L strains grow well on CT-SMAC and have high (256–1024 µg ml^−1^) Tel minimal inhibitory concentrations (MICs). S strains do not grow on CT-SMAC and have low (≤ 4 µg ml^−1^) Tel-MICs (Table 1). All five L strains contain all seven *ter* genes (*terZABCDEF*), whereas the corresponding S strains lack these genes (Table 1). These results were corroborated by Southern hybridization with a *terC* probe (data not shown).

**Table 1:** Characteristics of L and S colony variants of EHEC O157:H7 strains.

Strain no.^a^	Serotype	SF/GUD	Phage type	EHEC-Hly	Stx titre	*stx* genotype	*rfbE*_O157_	*fliC*_H7_	*ter*^b^	Tel-MIC (µg ml^−1^)	Growth on CT-SMAC^c^
81L	O157:H7	−/−	8	+	512	*stx*_2_	+	+	+	256	> 1000
81S	O157:H7	−/−	8	+	256	*stx*_2_	+	+	−	2	0
95L	O157:H7	−/−	8	+	256	*stx*_1_	+	+	+	512	> 1000
95S	O157:H7	−/−	8	+	256	*stx*_1_	+	+	−	4	1
134L	O157:H7	−/−	4	+	1024	*stx*_2_ + *stx*_2c_	+	+	+	256	> 1000
134S	O157:H7	−/−	4	+	512	*stx*_2_ + *stx*_2c_	+	+	−	2	0
135L	O157:H7	−/−	31	+	512	*stx*_2_	+	+	+	256	> 1000
135S	O157:H7	−/−	31	+	512	*stx*_2_	+	+	−	2	0
154L	O157:H7	−/−	8	+	128	*stx*_2c_	+	+	+	1024	> 1000
154S	O157:H7	−/−	8	+	256	*stx*_2c_	+	+	−	< 1	0

a L, large colony variant, and S, small colony variant of each strain indicated.

b All seven genes of the *ter* cluster (*terZABCDEF*) were tested; +, all were present; −, all were absent.

c Number of colonies grown after overnight incubation on plates inoculated with 1 × 10^5^ colony-forming units.

+, the characteristic was present; −, the characteristic was absent.

SF, sorbitol fermentation; GUD, production of β-D-glucuronidase; EHEC-Hly, EHEC haemolysin production; CT-SMAC, cefixime-tellurite sorbitol MacConkey agar.

### The *ter* cluster in L strains is located in homologues of OI 43/OI 48

**Fig. 2:**
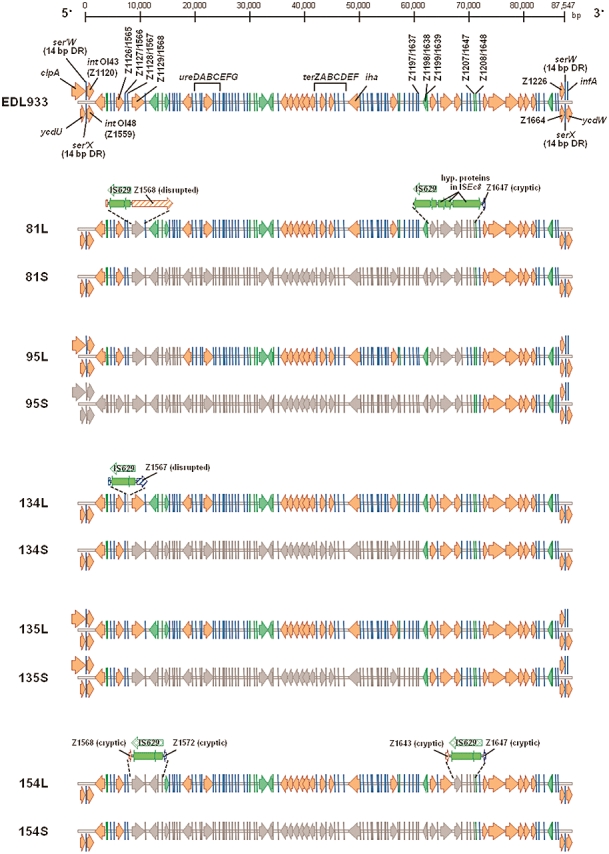
Tellurite resistance (Tel^R^) islands and flanking structures in L and S strains determined by PCR mapping. Single arrows indicate ORFs and flanks of OI 48 in strains that harbour only this OI. Duplicated arrows at the 5′ and 3′ ends of the island indicate the presence of two OI copies and depict the respective ORFs at these positions in OI 43 (upper arrows) and OI 48 (lower arrows). Genes located directly upstream (*clpA* and *ycdU*, respectively) and downstream (*serW/infA* and *serX/ycdW*, respectively) of OI 43 and OI 48 are also indicated. Orange arrows and blue lines indicate ORFs that were present and grey arrows/lines indicate absent ORFs. An ORF was considered present if an amplicon of the same size as that elicited from O157 strains EDL933 and Sakai was produced in the corresponding PCR. If no amplicon was produced, the ORF was considered absent (in strains with OI 43 and OI 48, only regions absent in both OIs could be identified as missing). ORFs with similarity to mobile genetic elements (putative transposases and insertion sequences) are highlighted in green. Insertion of novel IS elements is depicted above the corresponding regions in L strains (analysis of similar deletions in strain 95L was hindered by the presence of two Tel^R^–OI copies). Putative P4-family integrase genes (*int*), urease (*ure*) and tellurite resistance (*ter*) gene clusters, *iha* (encoding IrgA homologue adhesin), direct repeat regions (DR) flanking the OIs and intact/cryptic tRNA genes (*serW*/*ser'W* and *serX*/*ser'X*, respectively) are indicated. The scale (in bp) is above the graph. Results of PCR mapping of one of three independent S colonies derived from each L strain are shown (all three S derivates of the same L strain provided identical PCR results).

Analytical PCR (Fig. S1A, Table S1) demonstrated that all five L strains contain a complete *ter* cluster organized in the same order as in EDL933, and located within OI 43/OI 48 homologues (Fig. 2). However, in strains 81L, 134L and 154L we detected additional IS elements downstream and/or upstream of the *ter* cluster, which are not present in OI 43/OI 48 of EDL933 and which partially replaced island-specific genes (Fig. 2). Light cycler-based PCR demonstrated that strains 81L, 134L and 154L contain a single copy of Tel^R^-encoding island, while strains 95L and 135L contain duplicate copies, as in EDL933 (Fig. S2).

**Fig. 3:**
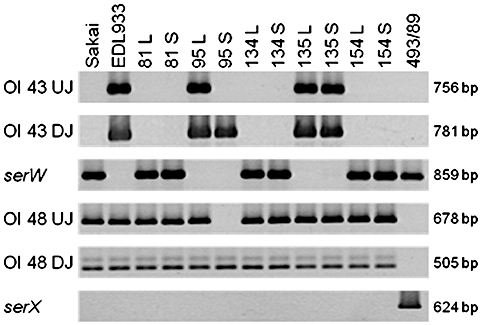
Amplification of *serW*, *serX* and the upstream (UJ) and downstream (DJ) junctions of OIs 43 and 48 in L and S variants of EHEC O157:H7 strains. Strains tested, PCR targets and lengths of PCR amplicons are listed across the top and to the left and right of the rows of amplicons respectively. Purified chromosomal DNA (20 ng) was used as a template in all PCRs. In PCRs targeting *serW* and *serX*, the presence of an amplicon of the same intensity as that from the positive control strain 493/89 (sorbitol-fermenting, *ter*-negative EHEC O157:NM that has intact *serW* and *serX*, as determined by sequence analysis in this study) indicates that the target locus is intact; the absence of an amplicon combined with amplification of UJ and DJ of the respective OI indicates that the locus is occupied by this OI. Amplification of UJs of OI 43 and OI 48 in strain 95S is hindered by the absence of the 5′ end of each OI in this strain (Fig. 2).

To identify the integration sites of the Tel^R^-encoding islands in L strains, we determined the intactness of tRNA genes *serW* and *serX* (Fig. S1B, Table S1), into which OI 43 and OI 48, respectively, are integrated in EDL933 (Perna *et al*., 2001). In strains 95L and 135L, *serW* and *serX* are occupied (Fig. 3), suggesting the presence of OI 43 and OI 48 homologues, respectively, in these locations. In strains 81L, 134L and 154L, *serX* is occupied and *serW* is intact (Fig. 3); these strains possess a homologue of OI 48 only. The synteny of these OIs and the two O157 genome reference strains was confirmed by PCRs targeting the upstream (5′) and downstream (3′) junction of each OI with the core genome (Fig. 3, Table 2) (designations of the 5′ and 3′ ends of OI 43/OI 48 used in this paper are based on the orientation of these OIs in the sequenced genome of EDL933; GenBank Accession No. AE005174).

**Table 2:** Copy numbers and genomic integration sites of Tel^R^-OI in L and S strains.

			Status of^c^		Genomic junctions of Tel^R^-OIs^d^			
						
Strain no.^a^	*ter*^b^	Tel^R^-OI copy no.	*serW*	*serX*	UJ/DJ OI 43	UJ/DJ OI 48	OI present	OI integrated in
81L	+	1	I	O	−/−	+/+	48	*serX*
95L	+	2	O	O	+/+	+/+	43 + 48	*serW, serX*
134L	+	1	I	O	−/−	+/+	48	*serX*
135L	+	2	O	O	+/+	+/+	43 + 48	*serW, serX*
154L	+	1	I	O	−/−	+/+	48	*serX*
81S	−	0	I	O	−/−	+/+	48T^e^	*serX*
95S	−	0	O	O	−/+	−/+	43T + 48T	*serW, serX*
134S	−	0	I	O	−/−	+/+	48T	*serX*
135S	−	0	O	O	+/+	+/+	43T + 48T	*serW, serX*
154S	−	0	I	O	−/−	+/+	48T	*serX*
EDL933^f^	+	2	O	O	+/+	+/+	43 + 48	*serW, serX*
Sakai^g^	+	1	I	O	−/−	+/+	48	*serX*
493/89^h^	−	0	I	I	−/−	−/−	None	n.a.

a L, large, and S, small colony variant of each respective strain.

b +, all seven genes of the *ter* cluster (*terZABCDEF*) were present; −, all seven genes were absent.

c I, the gene is intact; O, the gene is occupied.

d +, amplicon size was identical to that from strain EDL933; −, no amplicon was obtained.

e T, truncated OI 43 and/or OI 48.

f,g,h Control strains; data of PCR analyses are in agreement with complete genomic sequences of EHEC O157:H7 strains EDL933 (GenBank Accession No. AE005174), and Sakai RIMD 0509952 (GenBank Accession No. NC_002695); strain 493/89 (sorbitol-fermenting EHEC O157:NM) lacks the *ter* gene cluster (Bielaszewska *et al.*, 2005) and has *serW* and *serX* intact as determined by sequence analysis in this study.

Tel^R^-OI, tellurite resistance-encoding O islands; UJ, upstream (5′) junction; DJ, downstream (3′) junction; n.a., not applicable.

### S strains contain truncated homologues of OI 43/OI 48

All five S strains lack the *ter* gene cluster (Table 1), but except for missing 5′ ends of both islands in strain 95S, the junctions between OI 43 and/or OI 48 and the core genome are intact in all S strains (Figs 2 and 3, Table 2). Therefore, we systematically PCR-mapped (Fig. S1A) these truncated island(s) in three independent S colonies derived from each L strain to determine the extent of deletions. In all cases, the PCR suggested that a single deletion in an L strain resulted in the observed S colonies (representative S colony shown in Fig. 2). Moreover, in pulsed-field gel electrophoresis of XbaI-digested genomic DNA, all three S colonies that were descended from the same L strain shared identical restriction patterns, which differed by two to nine bands from that of the respective L strain (Fig. S3). This further confirmed that the genomic changes resulting from the deletions in OI 43/OI 48 were highly similar or identical in the derivatives of the same L strain. Altogether, the data from the PCR mapping and pulsed-field gel electrophoresis suggested that each of the parental L strains was ‘pre-programmed’ to undergo a particular sort of OI 43/OI 48 degeneration.

A precise characterization of the deleted regions is difficult in strains 95S and 135S, because both parental L strains contain the nearly identical (at least in EDL933) OI 43 and OI 48. In strain 135S, an internal portion of each OI of at least 52 kb was lost, and at least 70 kb of both islands were lost from strain 95S (Fig. 2). Moreover, the absence of *clpA* and *ycdU* (upstream of OI 43 and OI 48, respectively) from strain 95S (Fig. 2) suggests that a region of the upstream backbone genome was co-deleted with a major part of each island. Primer walking along the regions upstream of OI 43 and OI 48 (Table S2) demonstrated deletions of ∼ 2.9 kb (ORFs Z1118 and *clpA*) and ∼ 145.9 kb (ORFs Z1399 up to *ycdU*) of the core chromosome respectively. Analysis of a 3711 bp amplicon connecting ORFs Z1398 and Z1650, which spans the internal deletion of OI 48 as well as deleted parts of the core chromosome demonstrated that strain 95S lost in total a 217 535 bp fragment extending from the coding region of ORF Z1398 to the intergenic region between ORFs Z1646 and Z1647 (Fig. S4). Scrutiny of the respective ORFs/intergenic regions demonstrated no elements that could be responsible for homologous or site-specific recombination. We assume that a novel IS*629* integrated 596 bp downstream of the start codon of ORF Z1398, which rendered the gene cryptic, before the IS element recombined with a 259 bp cryptic IS*629* overlapping with ORF Z1647 (Fig. S4; see also next paragraph). Efforts to produce an amplicon spanning the deletion upstream of OI 43 failed.

The other three S strains harboured a truncated homologue of OI 48 that retained the 5′ end of the island from integrase gene (Z1559) to ORF Z1566 (∼ 7 kb; strain 134S) or Z1567 (∼ 8 kb; strains 81S and 154S), and the 3′ end of the island from ORF Z1638 (strain 134S) or Z1648 (strains 81S and 154S) to the last gene (Z1664) (∼ 26 and 16 kb, respectively) (Fig. 2). Large (∼ 53–62 kb) internal OI 48 regions were absent (Fig. 2).

### Analysis of deletions in truncated OI 48

**Fig. 4:**
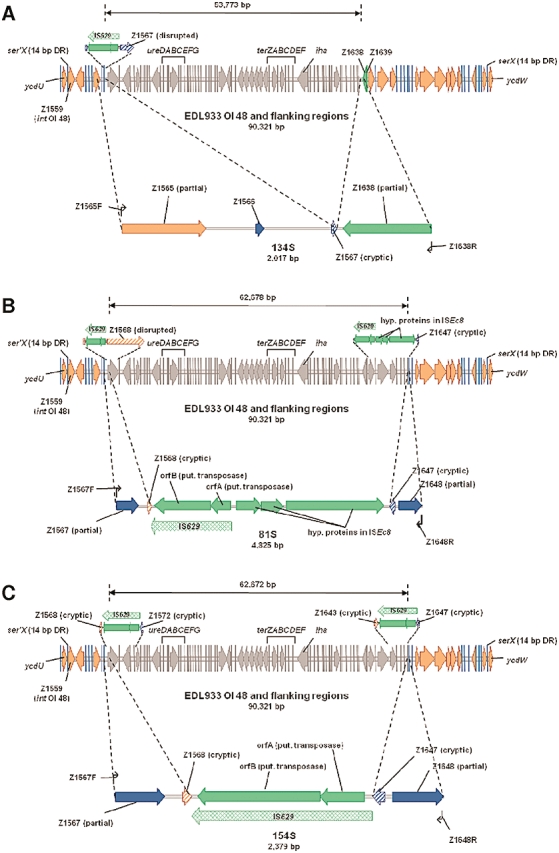
Deletions of OI 48 in strains 134S (A), 81S (B) and 154S (C). The upper part in each illustration shows the organization of OI 48 and its flanking regions in reference *E. coli* O157:H7 strain EDL933. Insertions of novel IS elements in the respective L strains are depicted above the island. The solid line above the genetic map depicts the extent of internal deletions in the three S strains (depicted in grey in the EDL933 sequence). In the lower part, the size (given in bp below the strain number) and genetic organization of the connecting fragment amplified after deletion of an internal part of OI 48 in each respective strain is shown (not drawn to scale). Small arrows symbolize primer binding sites for amplification of the connecting fragment (PCR primers and conditions are in Table S1). Outer dotted lines indicate homologies between OI 48 of EDL933 and the amplification product. The green arrows between the inner dotted lines depict sequences replacing the deleted region (IS, insertion sequence; hyp., hypothetical; put., putative). Indicated genes of OI 48 and flanking regions are *int* (putative P4-family integrase), *ure* (urease) and *ter* (tellurite resistance) clusters, *iha*, ORFs relevant to the deletion analyses, *serX*/*ser'X* (intact/cryptic tRNA gene) including direct repeats (DR) flanking the OI and *ycdU*/*ycdW* (chromosomal genes).

To determine the extent and putative mechanism(s) of deletions in OI 48 of the three strains with single *ter* islands, we PCR-amplified regions spanning the deletions in the S strains and their supposed boundaries in the corresponding L strains and sequenced the amplicons. In strain 134S, a 53 773 bp deletion extends from ORF Z1567 to the region between ORFs Z1637 and Z1638 (Fig. 4A). In the parental strain 134L we detected a full-length IS*629* element (1310 bp) that integrated 33 bp downstream of the start codon of ORF Z1567 (Figs 2 and 4A) and has 99% identity to ORFs Z1638/Z1639 (which have, in turn, 95% identity to IS*629* of EHEC O157 Sakai); this IS element is not present at this position either in EDL933 or O157 Sakai. Deletions in strains 81S (62 678 bp) (Fig. 4B) and 154S (62 672 bp) (Fig. 4C) start within ORF Z1568 and end in ORF Z1647, a putative transposase that overlaps with a 259 bp cryptic IS*629*. In strain 81L, a full-length IS*629* disrupted ORF Z1568 65 bp downstream of the start codon. In addition, ORFs Z1640 to Z1646 were substituted with a ∼ 3.7 kb mosaic structure composed of an additional IS*629* and ORFs similar to hypothetical proteins in IS*Ec8* (Figs 2 and 4B). In strain 154L, a full-length IS*629* occurs at the start point of the internal deletion between cryptic ORFs Z1568 and Z1572 replacing ORFs Z1569 to Z1571. At the end-point of the internal deletion we found a full-length IS*629* between cryptic ORFs Z1643 and Z1647 replacing Z1644 to Z1646 (Figs. 2 and 4C). Taken together, integration of novel IS elements into the Tel^R^-encoding islands of L strains and their subsequent homologous recombination with each other or with already existing IS elements such as ORFs Z1638/Z1639 and Z1647 (similar to IS*629*) caused the observed deletions (Fig. 4).

### Recombination between flanking direct repeats deletes Tel^R^-encoding OIs

In EDL933, OIs 43 and 48 are flanked by perfect 14 bp (TGGCGGTGAGGGGG) direct repeats (DRs), which encompass the last 14 bp of *serW* and *serX*, respectively, and are identical to the DRs of the *Shigella* Resistance Locus pathogenicity island (SRL-PAI) of *Shigella flexneri* 2a (Luck *et al*., 2001; Turner *et al*., 2004). Furthermore, each of these two OIs in EDL933 has an integrase gene that is 15 bp shorter than, but otherwise similar to, the integrase of the SRL-PAI (99% nucleotide identity). These observations suggest a mechanism whereby OI 43 and OI 48 could be lost by site-specific recombination between their DRs, similar to what is observed with SRL-PAI (Turner *et al*., 2001; 2004;) and other genomic islands (Rajanna *et al*., 2003; Middendorf *et al*., 2004; Sakellaris *et al*., 2004).

The five L and S *E. coli* O157:H7 strains were further analysed to test this hypothesis. Except for *int*_OI43_ of strain 95L and *int*_OI48_ of strain 135L, all other *int* genes in OIs 43 and 48 were identical in length and sequence to the corresponding genes in EDL933 (data not shown). *int*_OI43_ of 95L and *int*_OI48_ of 135L were of the same length as the *int* genes in EDL933, but contained six and seven mostly identical point mutations resulting in two and three amino acid changes, respectively (data not shown). Furthermore, in all but one of the L and S strains, the junctions between the core genome and the complete or truncated OI 43 and/or OI 48 are highly homologous (> 97%) to the boundaries of the corresponding OIs in EDL933 and contain the same 14 bp DRs. This suggests that both OI 43 and OI 48 could be deleted in their entirety by site-specific recombination. The only exception is strain 95S, which contains the 14 bp sequences corresponding to the DRs at the 3′ flank of OI 43 and OI 48, but the upstream junctions of these OIs and thus the 5′ DRs are missing (Figs 2 and 3); this makes complete excision of both OIs by site-specific recombination impossible.

Indeed, in strains 81L/81S, 95L, 134L/134S, 135L/135S and 154L/154S, which contain complete/truncated OI 43 and/or OI 48, intact *serW* and/or *serX* genes were amplified simultaneously with the upstream and downstream junctions of the islands using more template DNA (100 ng instead of 20 ng, data not shown). These dual amplicons indicate that a subpopulation of each outgrowth lost OI 43/OI 48 in their entirety. The same segment loss was observed in strains EDL933 and Sakai. Sequences of the intact *serW* and *serX* genes in each of these strains are identical to those in an OI 43- and OI 48-negative, *in silico*-generated derivative of EDL933 demonstrating that complete excision of OI 43 and/or OI 48 based on site-specific recombination between flanking DRs occurred in the strains. Taken together, these data demonstrate a complete excision of OI 43/OI 48, in addition to internal OI deletions, in *E. coli* O157:H7.

### Instability of Tel^R^-encoding OIs

The proportions of cells in overnight cultures that had sustained deletions were determined using quantitative real-time PCR. Complete and internal deletions of OI 48 occurred in 1.97 ± 0.75 × 10^−4^ (average data for all strains) and 9.70 ± 0.23 × 10^−1^ cells (average data for strains with OI 48 only) respectively. The complete excision of OI 43 occurred in a proportion of 1.81 ± 0.48 × 10^−3^ cells.

### Adherence of L and S strains to human intestinal epithelial cells and role of Iha

**Fig. 5:**
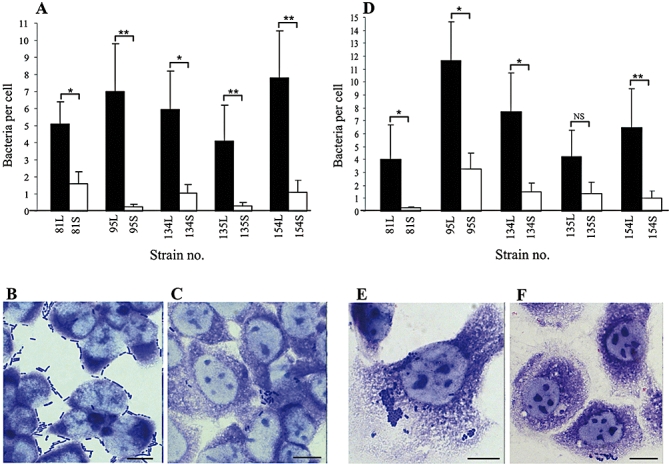
Adherence of *iha*^+^ L strains and their *iha*^−^ S derivatives to human intestinal epithelial cells. Strains were grown overnight in DMEM (iron < 0.05 µg ml^−1^) without shaking and adherence assay was performed as described in *Experimental procedures*. All bacteria and cells were counted in 10 randomly selected fields and bacteria per cell were averaged. Quantitative differences between adherence of the respective L and S strains were determined using unpaired Student's *t*-test. Morphological pattern of adherence was evaluated according to Scaletsky *et al*. (1999). A and D. Adherence of L and S strains to HCT-8 (A) and Caco-2 cells (D) quantified by numbers of bacteria attached per cell. *P < 0.05; **P < 0.001; NS, not significant. Data are expressed as mean ± standard deviations of number of bacteria attached per cell from three independent experiments. B, C, E and F. Adherence patterns on HCT-8 (B and C) and Caco-2 cells (E and F) exemplified by strain pair 95L (B and E) and 95S (C and F). Bars represent 10 µm.

We asked if deletions of OI 43 and OI 48 influence the capacity to adhere to human intestinal epithelial cells because these islands contain *iha*, encoding Iha (Fig. 2) (Tarr *et al*., 2000; Perna *et al*., 2001). We compared adherence of L (*iha*^+^) and S (*iha*^-^) strains to HCT-8 and Caco-2 cells using iron-limited conditions [Dulbecco's minimal essential medium (DMEM); iron < 0.05 µg ml^−1^] that significantly upregulate *iha* transcription compared with that in iron (10 µM FeCl_2_)-repleted DMEM (iron 0.50 µg ml^−1^) and Luria–Bertani (LB) broth (iron 0.59 µg ml^−1^) (Fig. S5). The DMEM-cultured L strains adhered significantly more efficiently than did their identically cultured *iha*^-^ S derivatives to HCT-8 cells, as demonstrated by numbers of bacteria attached per cell (Fig. 5A). Moreover, in most cases, the morphological pattern of the adherence differed between the L and S strains. L strains usually displayed diffuse adherence with most bacteria attaching to cell peripheries (Fig. 5B), whereas S strains adhered mostly in small loose clusters (Fig. 5C) in a localized adherence-like pattern (Scaletsky *et al*., 1999) or as scarce single bacteria. All DMEM-cultured L strains also adhered to Caco-2 cells to greater extents than did their S derivatives (Fig. 5D), but there was no distinct morphological difference between adherence of L and S strains; all strains displayed localized adherence-like patterns, with bacterial clusters being larger and more frequent in L (Fig. 5E) than in S strains (Fig. 5F). In contrast to the strains cultured in DMEM, no apparent difference in adherence to any of the cell lines was observed in any of the L/S pairs when strains were cultured in iron-repleted DMEM and in LB broth where minimal or no *iha* transcription occurs (Fig. S5). Under these conditions, all strains adhered weakly (range, 1–3, mean, 1.7 ± 0.8; and range, 0–4, mean, 1.8 ± 1.1 bacteria per cell respectively). These data suggest an important role of Iha in the adherence of L strains grown under iron-limited conditions to cultured intestinal epithelial cells.

We confirmed that the L and S strains contained the same panel of other proven or putative EHEC O157:H7 adhesins and determined their expression in DMEM. Each of the L and S strains harboured *eae* encoding intimin (Donnenberg *et al*., 1993), *lpfA1* and *lpfA2* encoding major fimbrial subunits of long polar fimbriae 1 and 2, respectively (Torres *et al*., 2002; 2004;), and *ehaA* encoding the EHEC autotransporter A (EhaA) (Wells *et al*., 2008). However, in contrast to *iha*, whose transcription in L strains is significantly upregulated in DMEM, relative transcription of the non-*iha* adhesin genes was low (usually below 2.0), and comparable in L and S strains (Fig. S6). Hence, Iha appears to be the major adhesin involved in the adherence of L strains to HCT-8 and Caco-2 cells under iron-limited conditions.

**Fig. 6:**
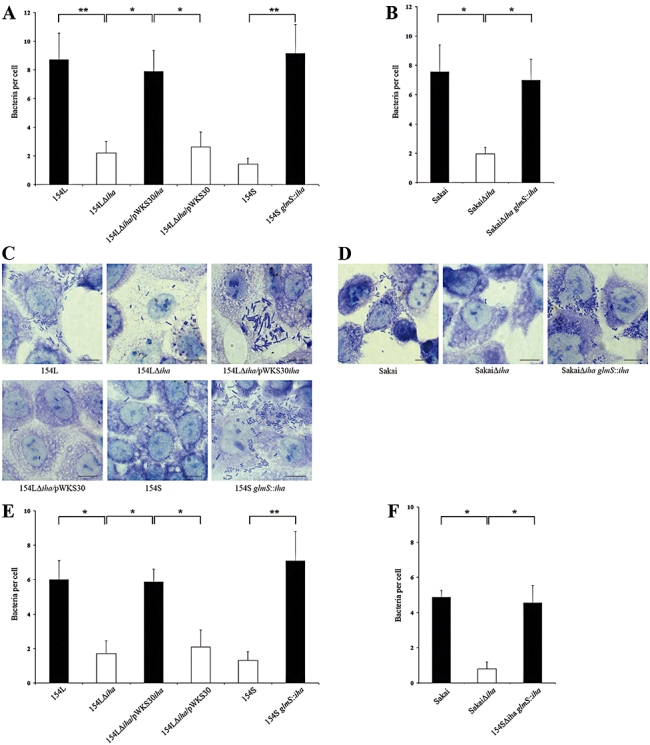
Role of Iha in the adherence of EHEC O157:H7 strains 154L and Sakai to human intestinal epithelial cells. Strains 154L, 154S and their *iha* deletion (154LΔ*iha*) and *iha* complementation (154LΔ*iha*/pWKS30*iha* and 154S *glmS*::*iha*) mutants (and 154LΔ*iha*/pWKS30 vector control) were grown overnight in DMEM without shaking and adherence assay was performed as described in *Experimental procedures*. EHEC O157:H7 strain Sakai, and its *iha* deletion (SakaiΔ*iha*) and *iha* complementation (SakaiΔ*iha glmS*::*iha*) mutants were tested in parallel. To quantify the adherence, all bacteria and cells were counted in 10 randomly selected fields and bacteria per cell were averaged. Differences between adherence of *iha*^+^ and *iha*^-^ strains were determined using unpaired Student's *t*-test. A, B, E and F. Adherence of wild-type strains and their *iha* deletion and *iha* complementation mutants to HCT-8 (A and B) and Caco-2 cells (E and F) quantified by numbers of bacteria attached per cell. *P < 0.05; **P < 0.001. Data are expressed as mean ± standard deviations of number of bacteria attached per cell from three independent experiments. C and D. Photomicrographs showing HCT-8 adherence patterns of strains analysed for quantitative adherence to these cells in A (C) and B (D). Bars represent 10 µm.

To further establish the role of Iha in adherence, we constructed an isogenic *iha* deletion mutant of strain 154L (154LΔ*iha*), and compared its adherence capacity with those of the parental strain 154L, *iha*-complemented 154LΔ*iha*, strain 154S and *iha*-complemented strain 154S (all cultured in DMEM) (for constructs see Table S4). The adherence capacity of the mutant 154LΔ*iha* to both cell lines was reduced nearly to the level of that of strain 154S (Fig. 6A, C and E). However, *iha* complementation of 154LΔ*iha* and of strain 154S restored the adherence capacity of each respective complemented strain (154LΔ*iha*/pWKS30*iha* and 154S *glmS*::*iha*) basically to that of strain 154L (Fig. 6A, C and E). To extend these findings, we compared the adherence of prototypic EHEC O157:H7 strain Sakai (RIMD 0509952), its isogenic *iha* deletion mutant (SakaiΔ*iha*) and *iha*-complemented deletion mutant (SakaiΔ*iha glmS*::*iha*). As in strain 154L, the *iha* deletion mutant of O157 Sakai adhered less well, whereas the *iha* complementation returned the adherence to the level of the wild-type strain (Fig. 6B, D and F). These experiments confirm a major contribution of Iha to the adherence of EHEC O157:H7 to cultured intestinal epithelial cells under iron-limited conditions. Notably, while completely restoring the adherence capacity quantitatively, the *iha* complementation of strains 154LΔ*iha* and 154S restored only partially (Fig. 6C) the peripheral adherence pattern produced by L strains on HCT-8 cells. This suggests that an additional bacterial or HCT-8 cell factor is involved in this adherence phenotype, which is specific for HCT-8 cells.

### Impact of Iha on the growth of L and S strains under iron-limited conditions

**Fig. 7:**
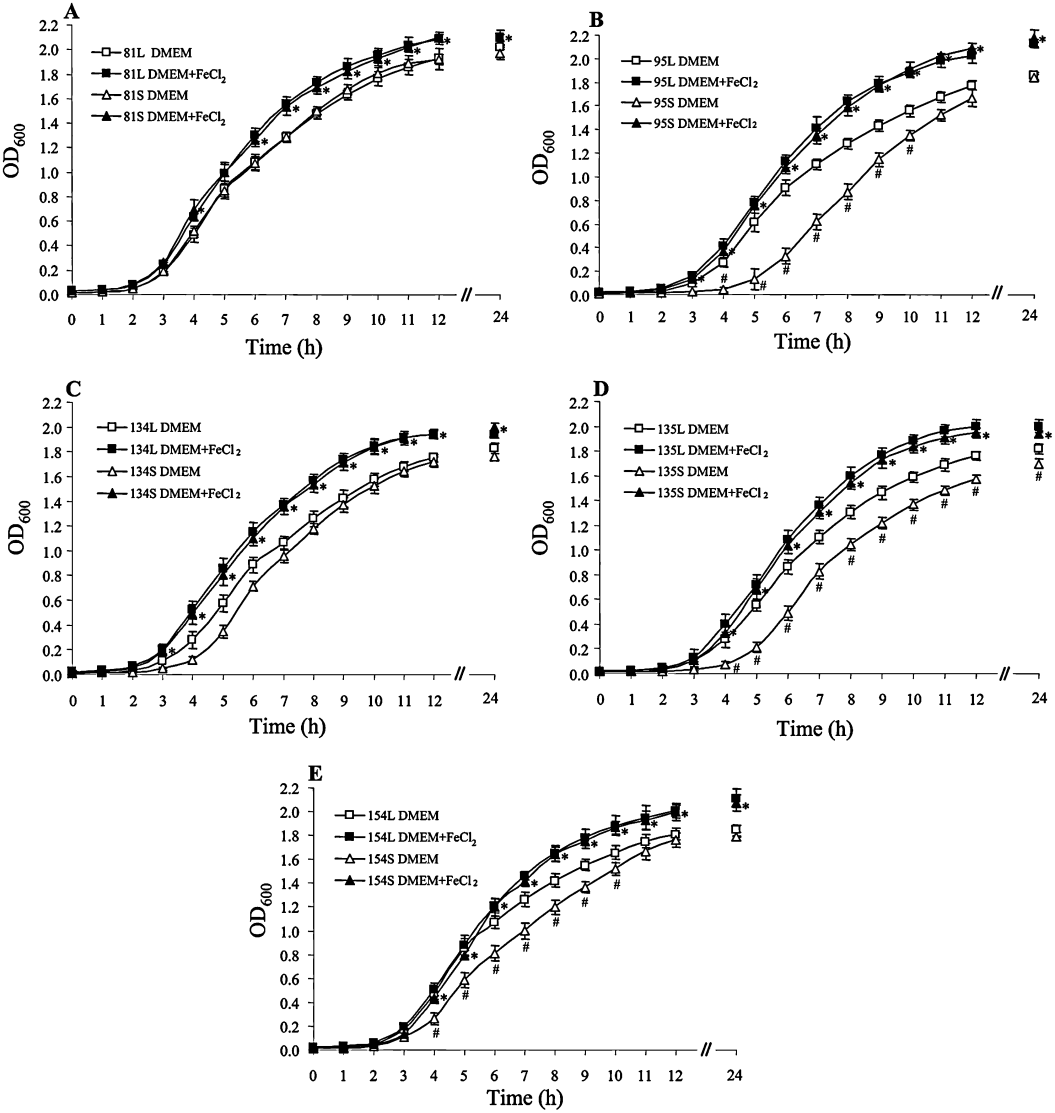
Growth of L (*iha*^+^) and S (*iha*^−^) variants of *E. coli* O157:H7 strains in iron-limited and iron-repleted conditions. A–E. L and S strains were grown in DMEM without (iron < 0.05 µg ml^−1^) and with 10 µM FeCl_2_ (iron 0.5 µg ml^−1^) and bacterial growth was monitored by measuring optical density at 600 nm (OD_600_) at the time points indicated. #, the difference in OD_600_ between the corresponding L and S strain grown in DMEM was statistically significant (*P* < 0.05; unpaired Student's *t-*test); *, the difference in OD_600_ between the S strain cultured in DMEM and in DMEM with 10 µM FeCl_2_, respectively, was statistically significant (*P* < 0.05; unpaired Student's *t-*test). Data are presented as means ± standard deviations from three independent experiments.

Because Iha is a siderophore receptor in uropathogenic *E. coli* (Léveillé*et al*., 2006), we asked if the *iha*^+^ L strains and their *iha*^-^ S derivatives grow differently under iron-limited conditions (DMEM) where *iha* transcription is upregulated (Fig. S5). In three of five strain pairs, L strains grew significantly more rapidly in DMEM than their corresponding S derivatives (Fig. 7). Repletion of DMEM with 10 µM FeCl_2_ remedied this growth impairment (Fig. 7). These data suggest that the lack of Iha contributes to reduced growth rates of S strains in low-iron-milieus.

**Fig. 8:**
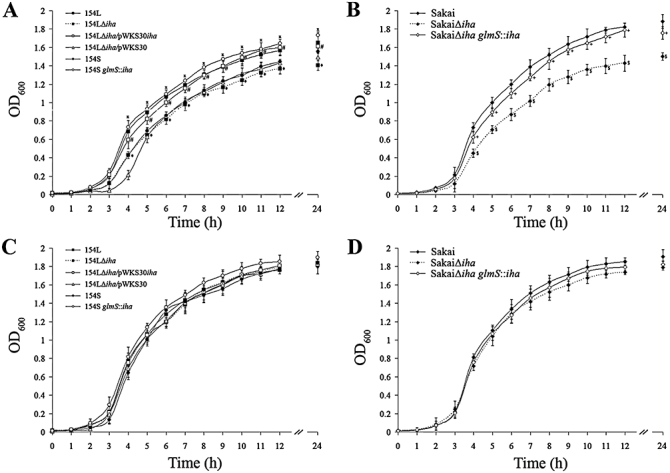
Impact of Iha on growth of EHEC O157:H7 strains 154L and Sakai under iron-limited and iron-repleted conditions. A–D. Strains 154L, 154S, and their respective *iha* deletion and *iha* complementation mutants 154LΔ*iha*, 154LΔ*iha*/pWKS30*iha*, and 154S *glmS*::*iha* (and 154LΔ*iha*/pWKS30 vector control) (A and C) and strains O157 Sakai, SakaiΔ*iha* and SakaiΔ*iha glmS*::*iha* (B and D) were grown in DMEM (A and B) and in DMEM with 10 µM FeCl_2_ (C and D) and bacterial growth was monitored by measuring OD_600_ at the time points indicated. The differences in OD_600_ values between 154L and 154LΔ*iha* (*), 154LΔ*iha* and 154LΔ*iha*/pWKS30*iha* (#), 154S and 154S *glmS*::*iha* (x), O157 Sakai and SakaiΔ*iha* (§), and SakaiΔ*iha* and SakaiΔ*iha glmS*::*iha* (+) grown in DMEM were statistically significant (*P* < 0.05; unpaired Student's *t-*test). Data are presented as means ± standard deviations from three independent experiments.

To test this hypothesis, we compared growth rates of strains 154L, 154S and their respective *iha* deletion and *iha* complementation mutants in DMEM and in DMEM with 10 µM FeCl_2_ (Fig. 8). In DMEM, the *iha* deletion mutant 154LΔ*iha* grew significantly slower than did the parental strain 154L, and comparably slow as did strain 154S. *iha* complementation of 154LΔ*iha* and 154S restored the growth rate of each respective complemented strain (154LΔ*iha*/pWKS30*iha* and 154S *glmS*::*iha*) to the level of strain 154L (Fig. 8A). The same impact of *iha* deletion and complementation on the growth in DMEM was observed in the O157 Sakai strain and its mutants SakaiΔ*iha* and SakaiΔ*iha glmS*::*iha* (Fig. 8B). Repletion of DMEM with 10 µM FeCl_2_ remedied the growth of *iha* deletion mutants 154LΔ*iha* and SakaiΔ*iha* (Fig. 8C and D), as also observed in strain 154S (Fig. 8C) and other S strains (Fig. 7). Taken together, these data suggest that Iha is essential for growth of EHEC O157:H7 under iron limitation and its absence in S strains impairs their growth under such conditions. The ability of S strains to grow, though slower, under iron deficiency can be explained by involvement of other (non-Iha) iron acquisition systems identified in EHEC (Torres and Payne, 1997; Kresse *et al*., 2007). The expression of such siderophore systems in all S strains is demonstrated by the ability of supernatants of overnight DMEM cultures to bind iron from a chrome azurol S/iron(III)/hexadecyltrimethylamonium bromide complex (Schwyn and Neilands, 1987) (data not shown).

### Influence of Iha on colony phenotype

**Fig. 9:**
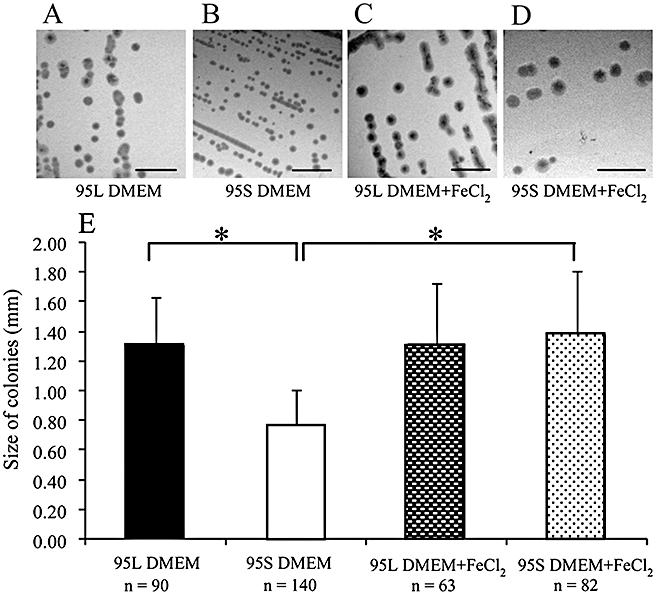
Influence of iron content on colony size of EHEC O157:H7 strains exemplified in strain pair 95L/95S. One L and one S colony of the strain was inoculated on a plate of DMEM agar and DMEM agar with 10 µM FeCl_2_ and incubated at 37°C for 26 h. After visual inspection, the plates were photographed and diameter of all or most well-separated colonies from each plate (*n*) was determined using a Power Point measuring tool. Differences between colony sizes under different conditions were calculated using unpaired Student's *t*-test. A–D. Size of L and S colonies of strain 95 cultured on DMEM agar without (A and B, respectively) and with 10 µM FeCl_2_ (C and D, respectively) as determined by visual inspection. Bars correspond to 5 mm. E. Size of L and S colonies on DMEM agar without and with 10 µM FeCl_2_ expressed as mean ± standard deviation of diameter of the indicated numbers (*n*) of colonies.*P < 0.05.

**Fig. 10:**
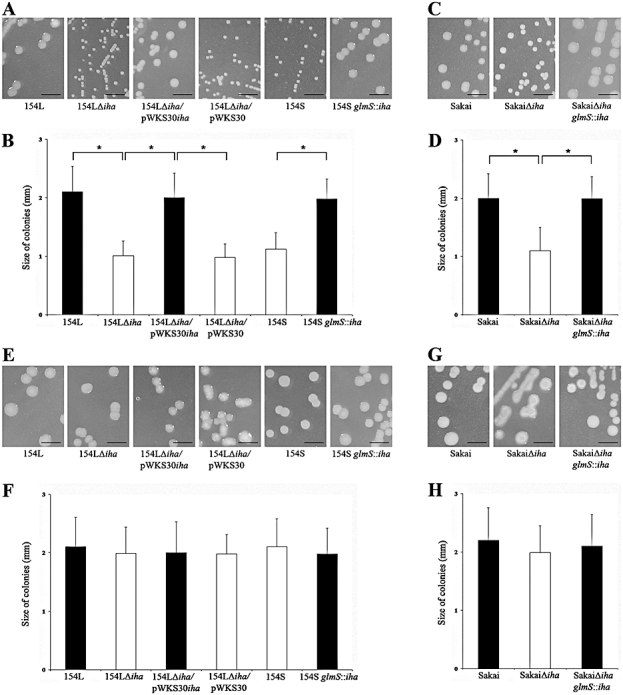
Influence of Iha on colony size. One colony of each strain 154L, 154S, O157 Sakai and their respective *iha* deletion and *iha* complementation mutants (as well as of 154LΔ*iha*/pWKS30 vector control) was inoculated on a plate of DMEM agar and DMEM agar with 10 µM FeCl_2_ and incubated at 37°C for 26 h. After visual inspection, the plates were photographed and diameter of at least 50 well-separated colonies was determined using a Power Point measuring tool. Differences between colony sizes of corresponding *iha*^+^ and *iha*^-^ strains were calculated using unpaired Student's *t*-test. A and C. Colony sizes of strains 154L, 154S and their respective *iha* deletion and *iha* complementation mutants (A) and of O157 Sakai strain and its *iha* deletion and *iha* complementation mutants (C) cultured on DMEM agar without FeCl_2_ as determined by visual inspection. Bars correspond to 5 mm. B and D. Colony sizes of the strains shown in A and C, respectively, expressed as mean ± standard deviation of diameter of at least 50 colonies.*P < 0.05. E and G. Colony sizes of strains 154L, 154S and their respective *iha* deletion and *iha* complementation mutants (E) and of O157 Sakai strain and its *iha* deletion and *iha* complementation mutants (G) cultured on DMEM agar with FeCl_2_ as determined by visual inspection. Bars correspond to 5 mm. F and H. Colony size of the strains shown in E and G, respectively, expressed as mean ± standard deviation of diameter of at least 50 colonies.

We further determined whether *iha* loss contributes to the reduced size of S colonies by comparing the sizes of S and the respective L colonies on DMEM agar without (iron < 0.05 µg ml^−1^) and with 10 µM FeCl_2_ (iron 0.50 µg ml^−1^). On plain DMEM agar, S colonies of all five strains were significantly smaller than their parental L colonies (example in Fig. 9A, B and E). Supplementation of DMEM agar with 10 µM FeCl_2_ significantly increased the size of S (example in Fig. 9D and E), but not L (Fig. 9C and E) colonies. Next, we compared colony sizes of strains 154L, 154S, and their respective *iha* deletion and *iha* complementation mutants grown as above. On DMEM agar, colonies of the 154LΔ*iha* mutant were significantly smaller than those of strain 154L, and of similar size to those of strain 154S (Fig. 10A and B). *iha* complementation of 154LΔ*iha* and 154S increased colony size of each respective complemented strain (154LΔ*iha*/pWKS30*iha* and 154S *glmS*::*iha*) to that of strain 154L (Fig. 10A and B). Similarly, *iha* deletion from the O157 Sakai strain significantly reduced colony size of the SakaiΔ*iha* mutant, whereas *iha* complementation of this mutant (SakaiΔ*iha glmS*::*iha*) returned the colony size to that of wild-type O157 Sakai (Fig. 10C and D). On DMEM agar with 10 µM FeCl_2_ colonies of all strains had similar sizes regardless of the presence or absence of *iha* (Fig. 10E–H). Thus, absence of Iha leads to atypical small colony phenotype in EHEC O157:H7, in particular on media with decreased iron content.

## Discussion

We are increasingly recognizing the non-static nature of the genomes of bacterial pathogens (Maurelli *et al*., 1998; Dobrindt *et al*., 2004; Brzuszkiewicz *et al*., 2006; Ahmed *et al*., 2008; Kaper and Karmali, 2008; Mellmann *et al*., 2009; Waldor, 2010). A rapid change in the genomic architecture of EHEC O157, even within a single strain, has been attributed to loss of *stx* genes and their encoding bacteriophages (Murase *et al*., 1999; Feng *et al*., 2001; Mellmann *et al*., 2005; 2008; Bielaszewska *et al*., 2006a). Such an event might enable strains to survive in the guts of humans and animals by avoiding lysis via *stx* phage induction (Mellmann *et al*., 2005; 2008;). Here, we demonstrate spontaneous loss of large internal regions of genomic islands OI 43 and/or OI 48 via homologous recombination between novel and existing IS elements, which removes Tel^R^ and Iha from clinical *E. coli* O157:H7 isolates. Additionally, complete excision of both islands is observed via site-specific recombination between flanking DRs in a proportion of cells, resembling site-specific excision of other genomic islands (Bach *et al*., 1999; Turner *et al*., 2001; Tauschek *et al*., 2002; Bueno *et al*., 2004; Middendorf *et al*., 2004; Sakellaris *et al*., 2004). In contrast, internal deletions of OI 48 occurred in a substantially higher proportion of cells (average of 9.7 × 10^−1^) than those reported for other genomic islands (10^−5^–10^−6^) (Turner *et al*., 2001; Tauschek *et al*., 2002; Middendorf *et al*., 2004). Nevertheless, the frequency is similar in magnitude to that observed for the ‘magnetosome island’ of *Magnetospirillum gryphiswaldense*, where spontaneous mutants affected in magnetosome formation arise at a frequency of up to 10^−2^ after prolonged storage or exposure to oxidative stress, a process assumed to be also based on integration of new IS elements and subsequent homologous recombination (Schübbe *et al*., 2003; Ullrich *et al*., 2005). Although we observed the deletions of OI 43 and/or OI 48 *in vitro* (i.e. during laboratory passage), isolation of Tel-susceptible EHEC O157:H7 directly from patients' stools (Bielaszewska *et al*., 2005) suggests that Tel^R^ island excisions also occur during infection.

Besides the implications for microbial diagnosis (i.e. Tel-susceptible EHEC O157:H7 will not grow on CT-SMAC), the deletions in OI 43/OI 48 might have consequences for virulence and evolution of EHEC O157:H7. Several lines of evidence support the role of OI 43/OI 48 in the virulence and/or fitness of EHEC O157:H7 and involvement of *iha* and *ter* gene cluster in this process. Tarr *et al*. (Tarr *et al*., 2000) demonstrated that EHEC O157:H7 strain 86–24 with a deletion in *iha* adhered less well than the parental strain with functional *iha* to HeLa cells, corroborating the role of Iha as an adhesin. In another study, deletion of *iha* from strain 86–24 reduced adherence of the mutant to pig enterocytes in an iron-restricted milieu of a ligated ileal loop, but not in an *in vitro* adherence assay using strains cultured in iron-rich brain heart infusion broth, in which Iha expression might have been compromised (Yin *et al*., 2009). Johnson *et al*. demonstrated the role of Iha in a murine model of uropathogenesis (Johnson *et al*., 2005). Our findings add to these data, by demonstrating that an *iha* deletion in strain 154L reduced adherence to cultured human intestinal epithelial cells under iron limitation, i.e. when *iha* is most robustly transcribed. However, this effect is not seen in iron repletion when little *iha* transcription occurs. We also report for the first time that *iha* can be lost spontaneously by wild-type EHEC O157:H7 strains via partial or full excision of OI 43/OI 48, and that this process reduces adherence to intestinal epithelial cells under iron limitation. Moreover, our ability to restore adherence capacity of strain 154S by *iha* complementation confirms that loss of *iha*, and not of neighbouring genes, reduced adherence of this strain (and likely also of the other S strains) to human intestinal epithelial cells. Our data strengthen the case for Iha as an iron-regulated adhesin of EHEC O157:H7. Moreover, the colonizing ability (and perhaps intestinal survival in general) of the *iha*^-^ derivatives might be further limited as a consequence of their decreased ability to compete for iron, as we demonstrated by reduced growth of *iha*^-^ S strains as well as *iha* deletions mutants 154LΔ*iha* and O157 SakaiΔ*iha* under iron-limited conditions. Whether or not this effect is associated with the proposed role of Iha, which is absent in S strains, as a siderophore receptor (Léveillé*et al*., 2006) warrants further investigations.

The functional role of *ter* genes in bacteria is not known. In *S. marcescens*, the *ter* genes on plasmid R478 encode, in addition to Tel^R^, resistance to pore-forming colicins (Whelan *et al*., 1995). Therefore, if the *ter* cluster in EHEC O157:H7 encodes a similar function, strains harbouring these loci might better compete in polymicrobial milieus. Consequently, the loss of the *ter* cluster might reduce virulence (or colonization capacity) because of diminished competitive potential. Our data do not allow us to evaluate the role of the *ter* cluster in adherence to human intestinal epithelial cells separate to that of the major contribution of Iha. However, currently available experimental data from our study and one other report (Yin *et al*., 2009) suggest that deletions within OI 43/OI 48 negatively influence virulence and/or fitness of EHEC O157:H7. This is of a particular importance considering the high frequency of such deletions.

Sorbitol-fermenting (SF) EHEC O157:NM (non-motile), a close relative of EHEC O157:H7 (Feng *et al*., 1998; Leopold *et al*., 2009), lacks complete or truncated OI 43/OI 48 integrated in *serW*/*serX* (Fig. 3). However, SF EHEC O157:NM strains possess a large mosaic island composed of fragments of SRL-PAI and ∼ 20 kb of the 3′ end of OI 43 of EDL933, which lacks *ter* and *iha* (Janka *et al*., 2005). Our finding of similar remnants of OI 43/OI 48 in Tel^S^ S variants of EHEC O157:H7 analysed in this study prompts speculation that SF EHEC O157:NM originally possessed a homologue of OI 43 that was subsequently truncated via genomic deletions and became a part of the mosaic island, probably during genomic rearrangements. Thus, deletions in OI 43/OI 48 might have played a role in the evolution of the EHEC O157 group. The finding of a hybrid island that contains segments of the 3′ end of OI 48 of EDL933 in EHEC O113:H21 (Shen *et al*., 2004) indicates the frequent occurrence of recombination events in this element. Moreover, a functional homologue of Tel^R^-encoding island is found in various non-O157 EHEC (Tarr *et al*., 2000) and in enterotoxigenic *E. coli* (Parreira *et al*., 2008) suggesting that this segment can be assimilated by divergent genomes.

Different scenarios might explain how Tel-susceptible EHEC O157:H7 (Taylor *et al*., 2002; Bielaszewska *et al*., 2005) arise. Such strains might have never had the Tel^R^-encoding islands. Alternatively, such strains might have originally possessed Tel^R^ island(s), which were subsequently completely excised from the chromosome by site-specific recombination, as demonstrated in our study. Indeed, intact *serW* and *serX* genes, potentially resulting from either of these scenarios, are found in some *E. coli* O157:H7 (Taylor *et al*., 2002). A third scenario based on our data, which appears to occur most frequently, is that Tel^S^ results from internal deletions in Tel^R^-encoding islands that encompass the *ter* gene cluster.

In summary, OI 43/OI 48 deletions are another mechanism of genome plasticity in the EHEC 1 clade. The deletions are accompanied by phenotypic and functional changes. These changes reduce virulence and/or fitness, and are at least partially attributed to the loss of *iha*, which might play dual roles in the virulence of EHEC O157:H7, i.e. as an adhesin and a siderophore receptor. The frequency of excision *in vivo*, the biologic role of this process and the survival consequences of these mutations warrant further investigation.

## Experimental procedures

### Bacterial strains and their genotypic and phenotypic characterization

The five *E. coli* O157:H7 strains displaying morphological dissociation associated with loss of Tel^R^-encoding islands were isolated during 6 years from five patients (four with haemolytic uraemic syndrome and one with bloody diarrhoea) living in five different cities in Germany, indicating epidemiological independence between the strains. The dissociation into L and S colonies was observed after two to four passages on SMAC agar (Becton Dickinson, Sparks, MD, USA). Between these passages the strains were stored between 1 and 5 days at 4°C. L and S colonies from each strain were biochemically confirmed as *E. coli* (API 20 E; bioMérieux, Marcy l'Etoile, Lyon, France), serotyped (Prager *et al*., 2003), phage typed (Liesegang *et al*., 2000) and tested by PCR for *rfbE*_O157_ (Nagano *et al*., 1998), *fliC*_H7_ (Eklund *et al*., 2006), *stx* genotype (Friedrich *et al*., 2002; Bielaszewska *et al*., 2006b) and the *terZABCDEF* cluster (Bielaszewska *et al*., 2005). Tel-MICs were determined using microdilution (Sahm and Washington, 1991). Each strain was tested in duplicate and in two independent experiments using 5 × 10^4^ cfu per well and serial dilutions (from 1024 to 1 µg ml^−1^) of potassium tellurite (K_2_TeO_3_) (Sigma-Aldrich, Taufkirchen, Germany) in 100 µl of LB broth. The MIC was defined as the lowest concentration of K_2_TeO_3_ that completely inhibited growth after overnight incubation at 37°C. The ability to grow on CT-SMAC agar (K_2_TeO_3_ 2.5 µg ml^−1^, cefixime 0.05 µg ml^−1^; Becton Dickinson) was determined on plates inoculated with 1 × 10^5^ cfu after overnight incubation (Bielaszewska *et al*., 2005). Stx titres were determined in a Vero cell assay (Bielaszewska *et al*., 2006b) and defined as the reciprocal of the highest dilution of culture supernatant that was cytotoxic in 50% of cells after 3 days of incubation. Production of EHEC haemolysin was sought on enterohaemolysin agar (Sifin, Berlin, Germany) and β-D-glucuronidase activity was assessed using nutrient agar with 4-methylumbelliferyl-β-D-glucuronide (MUG) (Becton Dickinson).

### PCR assays for mapping and analyses of deletions of Tel^R^-encoding islands

Polymerase chain reaction primers and conditions are listed in Table S1. Positions of the PCR primers in OI 43/OI 48 of *E. coli* O157:H7 strain EDL933 and the flanking regions are depicted in Fig. S1 and primers used to analyse internal deletions in OI 48 are depicted in Fig. 4. PCRs for mapping of Tel^R^-encoding islands were performed in the iCycler (version 1.259; Bio-Rad, München, Germany) using reagents from PEQLAB Biotechnologie (Erlangen, Germany) (Sonntag *et al*., 2004) and 2.5 µl of bacterial DNA purified with InstaGene Matrix (Bio-Rad) as a template. PCRs to detect integration sites of Tel^R^-encoding islands, junctions between OI 43/OI 48 and the core genome, and to produce connecting fragments for sequence analysis of OI 43/OI 48 deletions were performed in a Biometra thermocycler using the RED Taq ReadyMix PCR Reaction Mix with MgCl_2_ (Sigma-Aldrich, München, Germany). The PCR master mix (20 µl) contained 20–100 ng of chromosomal DNA as a template and 10 pmol of each primer. Six microlitre aliquots of the reactions were analysed by electrophoresis in 1% (wt/vol) agarose gels. *E. coli* O157:H7 strain EDL933 (Perna *et al*., 2001), *E. coli* K-12 strain MG1655 (Blattner *et al*., 1997) and SF EHEC O157:NM strain 493/89 (Janka *et al*., 2005) were used as PCR controls.

### Analysis of the core genome deletions in strain 95S

The extents of the core genome deletions upstream of OI 48 and OI 43, respectively, in strain 95S were investigated using primer walking along each respective region starting from *ycdU* and *clpA*, respectively (for PCR primers see Table S2). Connecting fragment spanning deletion upstream of OI 48 was produced using primers Z1398-1 and Z1650-2 (Tables S2 and S1, respectively) and sequenced as described below. To produce a connecting fragment spanning the deletion upstream of OI 43, ORFs Z1117, Z1116 and Z1115 found to be present upstream of OI 43 using the primer walking were PCR connected (primers Z1117-1, Z1116-1 and Z1115-1, respectively) (Table S2) with ORFs Z1210 and Z1211 downstream of the internal deletion in OI 43 (Fig. 2); primers Z1650-2 and Z1651-2 (Table S1) that target the identical ORFs Z1210 and Z1211, respectively, in OI 43 were used for this purpose.

### Sequence analysis

Amplicons were sequenced using purified PCR products (PCR Purification Kit; Qiagen, Hilden, Germany), and an automated ABI Prism 3130xl Genetic Analyzer and the ABI Prism BigDye Terminator Ready Reaction Cycle Sequencing Kit (version 3.1, Applied Biosystems, Darmstadt, Germany). Sequences were analysed using the Vector NTI Advance 11 software (Invitrogen, Karlsruhe, Germany). Homology searches were performed using the EMBL-GenBank database (http://www.ncbi.nlm.nih.gov/BLAST).

### Light cycler-based PCR quantification of *terC*

Genomic DNA was isolated using the DNeasy Kit (Qiagen). *terC* and *gyrB* (used as an internal standard) were amplified using the QuantiTect SYBR Green PCR Kit (Qiagen) and primer pairs TerC-F1/TerC-R1 and GyrB-F2/GyrB-R2, respectively (Taylor *et al*., 2002). The PCRs were performed in the LightCycler System (Roche Diagnostics, Mannheim, Germany) as described (Zhang *et al*., 2005). After the final cycle, a melting curve analysis was performed with continuous fluorescence reading from 65°C to 95°C. A standard curve for the determination of DNA concentration was prepared using 10-fold dilutions of the total genomic DNA from *E. coli* O157:H7 strain Sakai (RIMD 0509952) ranging from 10^−1^ (20 ng µl^−1^) to 10^−5^ (2 pg µl^−1^). The concentrations of *terC* and *gyrB* DNAs were determined using LightCycler Software 3 second derivative method analysis (Roche Diagnostics) and *terC* DNA was normalized to *gyrB* DNA. The *terC*/*gyrB* DNA ratio for each strain was expressed as a mean (standard deviation) of three independent experiments.

### Quantitative real-time RT-PCR

Total RNA was isolated from L and S strains grown in LB broth and DMEM using the RNeasy Mini Kit (Qiagen). Co-purified DNA was removed using RNase-free DNase (Roche Diagnostics). A one-step quantitative real-time RT-PCR, performed with an iCycler iQ-5 (Bio-Rad) and the QuantiTect SYBR Green RT-PCR kit (Qiagen) measured the relative expression of mRNA of *iha*, *eae*, *lpfA1*, *lpfA2* and *ehaA*. The PCR reactions were performed in 96-well plates using a 20 µl volume containing 1 µl of total RNA (100 ng), 10 µl of 2× QuantiTect SYBR Green RT-PCR master mix, 0.2 µl of QuantiTect RT mix and 200 nM of each primer (Blumer *et al*., 2005; Léveillé*et al*., 2006; Chen *et al*., 2007; Torres *et al*., 2009) (for primers see Table S3). The PCR included a reverse transcription step at 50°C for 30 min, and polymerase activation and preliminary denaturation at 95°C for 15 min, followed by 35 cycles of denaturation at 94°C for 10 s, annealing at 53°C to 60°C for 20 s and extension at 72°C for 20 s. A melting curve analysis to confirm the specificity of the amplification products was constructed with continuous fluorescence reading from 55°C to 95°C. Data were analysed using the Bio-Rad iQ5 standard edition optical system software V2.0. The *iha*, *eae*, *lpfA1*, *lpfA2* and *ehaA* mRNAs were normalized to *gapA* mRNA. Each PCR was performed three times with three independent RNA preparations.

### Determination of deletions affecting the Tel^R^-encoding island

A real-time PCR approach with the StepOnePlus Real-Time PCR System (Applied Biosystems) was used to determine the proportion of intact *serW* and *serX* tRNA genes resulting from site-specific excision of OI 43 and OI 48, respectively, and of internal deletions in OI 48 in DNA extracted (DNAeasy kit; Qiagen) from overnight cultures of reference strains EDL933, Sakai, 493/89 as well as all L and S strains and adjusted to a concentration of 10 ng µl^−1^. All reactions were run in triplicate for 40 cycles and contained a mixture of 2–4 µl chromosomal DNA (10 ng µl^−1^), 2 µl of each primer (5 pmol µl^−1^) and 1xSYBR Green PCR Master Mix in a total volume of 20 µl according to the manufacturer's instructions. Post-experimentally, a melting curve analysis was performed (60°C to 95°C with 0.3°C increments) to verify product purity. All data were analysed with the StepOne Software v2.1.

To determine the amount of genome equivalents (GE) per µl of DNA solution (#*GE*), primer pair 131 (Table S1) was used to amplify an internal fragment of *recA* from all strains. As a standard 10^2^–10^6^ GEs of strain Sakai were used (calculation based on the published genome size) (Hayashi *et al*., 2001). Subsequently, 10^2^–10^6^ copies of strain 493/89, an OI 43/OI 48-negative derivative, were used as standard for PCRs 125 and 126, respectively (Table S1), to determine the amount of GEs with intact *serW* (*W_i_*) and *serX* (*X_i_*) genes. The proportion of cells with full excision of the respective island, i.e. OI 43-negative (*43^neg^*) and/or OI 48-negative (*48^neg^*), was calculated as the quotient of *W_i_* and #*GE* and *X_i_* and #*GE* respectively. Finally, an equimolar mixture of 10^2^–10^6^ copies of plasmid pTerE (3417 bp) (Table S4) and GEs of strain 493/89 was used as a standard in PCR 132 (Table S1) to amplify an internal fragment of *terE* from strains Sakai, 81L, 134L and 154L (each contains only a single Tel^R^-encoding island) and determine the amount of *terE*-positive GEs (*E^+^*). The proportion of GEs with internal deletions of OI 48 (*E^int−^*) was calculated according to the following formula: *E^int−^* = [(#*GE* − *E^+^*) − *48^neg^*]/#*GE*.

### Southern blot hybridization

Genomic DNA was digested with BamHI and PstI (New England Biolabs, Frankfurt, Germany), separated in 0.6% agarose and transferred to a nylon membrane. The membrane was probed under stringent conditions with a digoxigenin-labelled *terC* probe (Taylor *et al*., 2002) using DIG DNA Labelling and Detection Kit (Roche Diagnostics) (Bielaszewska *et al*., 2005).

### Pulsed-field gel electrophoresis

Pulsed-field gel electrophoresis was performed using the PulseNet protocol (Hunter *et al*., 2005) except that the running time was prolonged to 40 h to achieve more distinct separation of smaller bands. The XbaI-digested DNA of *Salmonella enterica* serovar Braenderup strain H9812 was used as a standard (Hunter *et al*., 2005). Restriction patterns were analysed and the cluster analysis was performed with BioNumerics software, version 5.1 (Applied Maths BVBA, Sint-Martens-Latem, Belgium).

### Construction of *iha* deletion and complementation mutants

The *iha* deletion mutants of EHEC O157:H7 strains 154L (154LΔ*iha*) and Sakai (RIMD 0509952) (SakaiΔ*iha*) were generated using lambda red-based recombineering (Datsenko and Wanner, 2000). Briefly, the chloramphenicol acetyltransferase gene (*cat*) cassette of plasmid pKD3 was amplified using primers del_iha_for and del_iha_rev (Table S5), with overhangs homologous to the 5′ and 3′ regions of the O157 Sakai *iha* gene. Purified PCR product was transformed into electrocompetent O157 Sakai or 154L cells carrying the plasmid pKD46. The *cat* cassette was cured upon transformation with plasmid pCP20 (Cherepanov and Wackernagel, 1995). *iha* mutants were screened using PCR and Southern blot.

For *in trans* complementation of *iha* mutants, a 2916 bp genomic fragment of strain O157 Sakai that contained the functional *iha* gene including its 400 bp upstream and 350 bp downstream region was amplified by PCR using the Phusion DNA polymerase (New England Biolabs) and the primers iha_for2 and iha_rev (Table S5). Purified PCR product was ligated into plasmid pWKS30 (Wang and Kushner, 1991) that had been linearized by restriction with SmaI and dephosphorylated with Antarctic phosphatase (both New England Biolabs). Screening for correct plasmid clones (pWKS30*iha*) was performed by PCR and the correct orientation of the insert was verified by sequencing. Strain 154LΔ*iha* was transformed with pWKS30 and pWKS30*iha* respectively.

For chromosomal complementation of *iha* mutants, the functional *iha* gene including its 400 bp upstream and 350 bp downstream region was PCR-amplified using primers iha-pKD4_for and iha-pKD4_rev (Table S5). Following digestion with BstBI/HindIII (New England Biolabs), the resulting 2916 bp PCR product was purified and ligated into BstBI/HindIII-digested plasmid pKD4. Screening for correct plasmid clones (pKD4*iha*) was performed using PCR and verified by sequencing. The *iha* fragment together with the kanamycin resistance gene was then PCR-amplified using the Phusion DNA polymerase and primers iha-int_for and iha-int_rev (Table S5). The resulting 3971 bp PCR product was transformed into relevant *iha* mutants carrying plasmid pKD46. Selection of transformants in which the *iha*::*kan* fragment was chromosomally inserted downstream *glmS* was done on LB agar plates supplemented with kanamycin (30 µg ml^−1^). Screening for strains SakaiΔ*iha* and 154S chromosomally complemented with *iha* was made by PCR and correct insertion of the *iha*::*kan* fragment was verified by sequencing. *iha* transcription in all *iha^+^* constructs was confirmed by quantitative real-time RT-PCR as described above.

### Iron content in culture media

Iron content in culture media was determined using atomic absorption spectroscopy. Briefly, LB broth, DMEM (Johnson *et al*., 2005) and DMEM supplemented with 10 µM FeCl_2_ were complemented with 1 ml of HNO_3_ per 100 ml. A triplicate of each solution was analysed in a Unicam Solaar 939 AA spectrometer with acetylene/air burner (split 10 cm) at 248.3 nm. Final iron content was calculated using a linear external calibration (0.1, 0.3, 0.5, 0.7, 0.9, 1.1, 1.3, 1.5 mg l^−1^) (DIN 38406–32, 2000). Based on this analysis, the media contained 0.59 µg ml^−1^, < 0.05 µg ml^−1^ and 0.50 µg ml^−1^ of iron respectively. DMEM agar and DMEM agar with 10 µM FeCl_2_ were prepared from liquid media by adding 1.5% (wt/vol) of agar–agar base (Carl Roth, Karlsruhe, Germany).

### Cell cultures and adherence assay

Human ileocaecal adenocarcinoma epithelial cell line HCT-8 (ATCC CCL-244) and colonic carcinoma cell line Caco-2 (German collection of microorganisms and cell cultures, Braunschweig, Germany; ACC 169) were cultured as described (Sonntag *et al*., 2005; Aldick *et al*., 2007). For adherence assays, 10^5^ cells per well were seeded in 24-well plates (Corning, Corning, NY, USA) containing coverslips and grown until they were ∼ 70% confluent. The cells were washed with phosphate-buffered saline (PBS), replenished with fresh medium with 0.5% D-mannose (Merck, Darmstadt, Germany), and infected with ∼ 1 × 10^8^ cfu of overnight stationary cultures of L and S strains, O157 Sakai strain and their respective *iha* deletion and *iha* complementation mutants (Table S4) in DMEM, DMEM with 10 µM FeCl_2_ or LB broth (only L and S strains were grown in the latter two media). After 3 h of incubation with bacteria (37°C, 5% CO_2_), cells were washed three times with PBS, and incubated another 3 h in fresh culture medium. The cultures were 10 times washed with PBS, fixed (70% ethanol), stained (10% Giemsa) (Merck, Darmstadt, Germany) and mounted using Glycergel (DakoCytomation, Hamburg, Germany). Bacterial adherence was examined using light microscopy (Axio Imager A1; Zeiss, Jena, Germany) and the adherence patterns were photographed (AxioCam MRm camera) (Zeiss). Bacteria and cells were counted in 10 randomly selected fields on each coverslip and bacteria per cell were averaged. The enumerator was unaware of the identity of the cells being counted. Differences in quantitative adherence of *iha*^+^ and *iha*^-^ strains were evaluated using unpaired Student's *t*-test (*P* < 0.05 considered significant).

### Growth in DMEM

One colony of each L strain, S strain, O157 Sakai strain and the respective *iha* deletion and *iha* complementation mutants (Table S4) was grown overnight (37°C, 180 r.p.m.) in 2 ml of DMEM without or with 10 µM FeCl_2_. An aliquot of the overnight culture was inoculated in 20 ml of the same medium to produce an OD_600_ between 0.015 and 0.025 (the starting OD_600_ values of corresponding L and S strains and the respective *iha* mutants were identical). Bacterial growth (37°C, 180 r.p.m.) was monitored spectrophotometrically (OD_600_) hourly for 12 h and again at 24 h. Each strain was tested in each medium in triplicate and growth curves were constructed by plotting mean OD_600_ values (standard deviations) against time. Differences in growth kinetics of *iha*^+^ and *iha*^-^ strains were evaluated using unpaired Student's *t*-test.

### Influence of iha expression on colony size

One colony of each L strain, S strain, the O157 Sakai strain and their corresponding *iha* deletion and *iha* complementation mutants (Table S4) was inoculated on DMEM agar without or with 10 µM FeCl_2_. After incubation at 37°C for 26 h the plates were photographed and the sizes of all or most well-separated colonies on each plate were determined using a Power Point (Microsoft) measuring tool. Differences between sizes of *iha*^+^ and *iha*^-^ colonies under different conditions were calculated using unpaired Student's *t*-test.

### Siderophore expression

Siderophore expression was detected colorimetrically in supernatants of overnight cultures of S strains grown in DMEM using chrome azurol S/iron(III)/hexadecyltrimethylamonium bromide complex as an indicator of iron binding (Schwyn and Neilands, 1987).
