# Supplementary material for: Chromosomal instability in enterohaemorrhagic Escherichia coli O157:H7: impact on adherence, tellurite resistance and colony phenotype
Source: Mol Microbiol. 2011 Jan 4;79(4):1024–44. doi: 10.1111/j.1365-2958.2010.07499.x (PMC3064760; doi:10.1111/j.1365-2958.2010.07499.x)
Supplement: Supplementary file 1 [file mmi0079-1024-SD1.pdf]

Supporting Information

Chromosomal Instability in Enterohaemorrhagic *Escherichia coli* O157:H7: Impact on Adherence, Tellurite Resistance and Colony Phenotype

Martina Bielaszewska[1,2]*[#], Barbara Middendorf[1#], Phillip I. Tarr[3], Wenlan Zhang[1], Rita Prager[4], Thomas Aldick[1], Ulrich Dobrindt[1,5], Helge Karch[1,2], and Alexander Mellmann[1,2]

[1] Institute of Hygiene and the National Consulting Laboratory on Haemolytic Uraemic Syndrome, University of Münster, Robert Koch Str. 41, 48149 Münster, Germany

[2] Interdisciplinary Centre of Clinical Research (IZKF) Münster, University of Münster, Domagkstr. 3, 48149 Münster, Germany

[3] Division of Paediatric Gastroenterology and Nutrition, Edward Mallinckrodt Department of Paediatrics, Washington University School of Medicine and St. Louis Children's Hospital, 660 South Euclid Avenue, St. Louis, MO 63110, USA

[4] National Reference Centre for Salmonella and Other Bacterial Enteric Pathogens, Robert Koch Institute, Branch Wernigerode, Burgstr. 37, 38855 Wernigerode, Germany

[5] Institute for Molecular Infection Biology, University of Würzburg, Josef-Schneider-Str. 2, 97080 Würzburg, Germany

[#] Both authors contributed equally

*Corresponding author:
Dr. Martina Bielaszewska, Institut für Hygiene, Universität Münster, Robert Koch Str. 41, 48149 Münster, Germany
Phone: ++49-251-980-2849; Fax: ++49-251-980-2868; E-mail: mbiela@uni-muenster.de

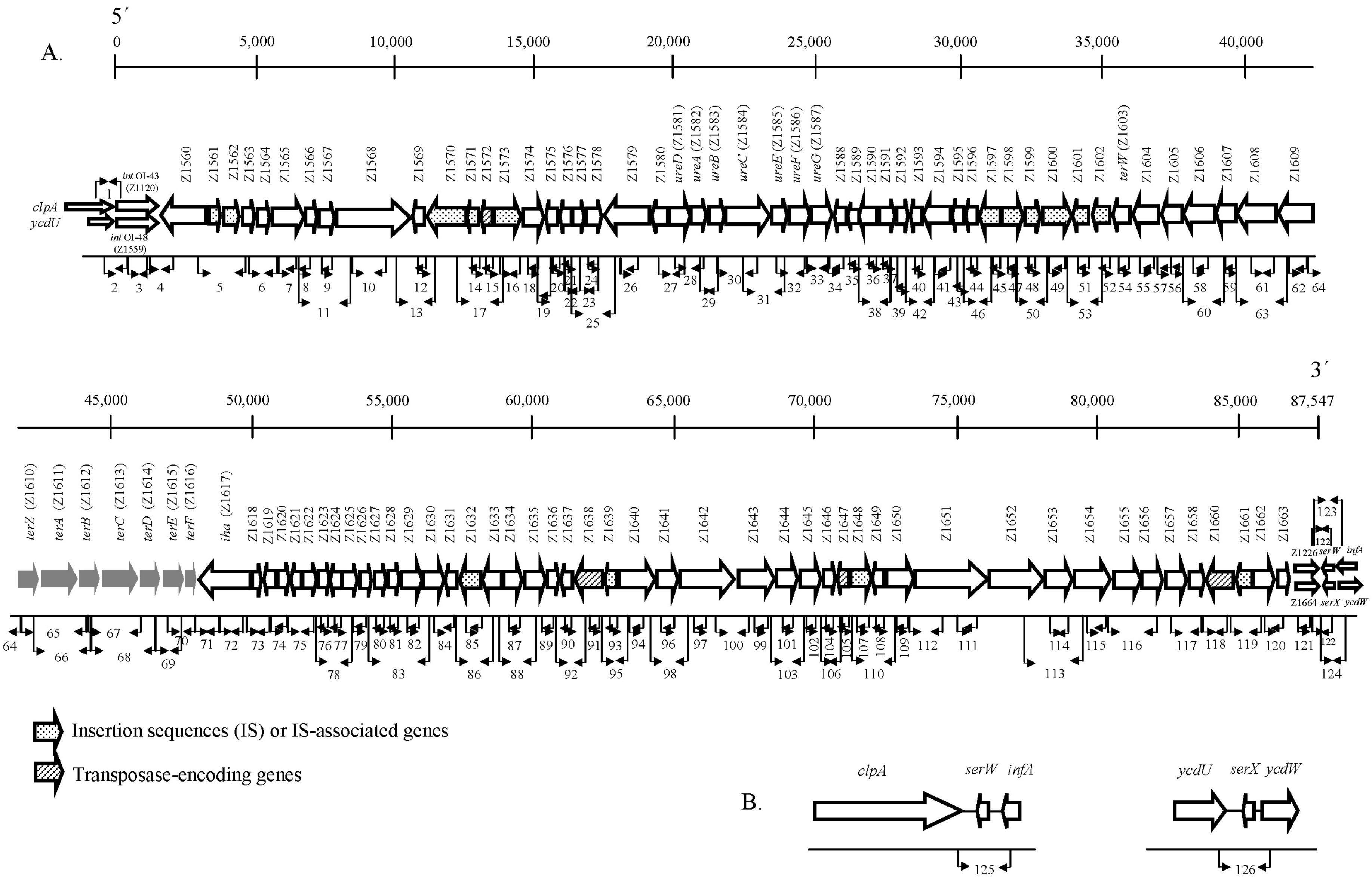
A.
5′
0
5,000
10,000
15,000
20,000
25,000
30,000
35,000
40,000
clpA
ycdU
int OI-43 (Z1120)
int OI-48 (Z1559)
Z1560 Z1561 Z1562 Z1563 Z1564 Z1565 Z1566 Z1567 Z1568 Z1569 Z1570 Z1571 Z1572 Z1573 Z1574 Z1575 Z1576 Z1577 Z1578 Z1579 Z1580
ureD (Z1581) ureA (Z1582) ureB (Z1583) ureC (Z1584) ureE (Z1585) ureF (Z1586) ureG (Z1587)
Z1588 Z1589 Z1590 Z1591 Z1592 Z1593 Z1594 Z1595 Z1596 Z1597 Z1598 Z1599 Z1600 Z1601 Z1602
terW (Z1603)
Z1604 Z1605 Z1606 Z1607 Z1608 Z1609
3′
45,000
50,000
55,000
60,000
65,000
70,000
75,000
80,000
85,000
87,547
terZ (Z1610) terA (Z1611) terB (Z1612) terC (Z1613) terD (Z1614) terE (Z1615) terF (Z1616)
iha (Z1617)
Z1618 Z1619 Z1620 Z1621 Z1622 Z1623 Z1624 Z1625 Z1626 Z1627 Z1628 Z1629 Z1630 Z1631 Z1632 Z1633 Z1634 Z1635 Z1636 Z1637 Z1638 Z1639 Z1640 Z1641 Z1642 Z1643 Z1644 Z1645 Z1646 Z1647 Z1648 Z1649 Z1650 Z1651 Z1652 Z1653 Z1654 Z1655 Z1656 Z1657 Z1658 Z1660 Z1661 Z1662 Z1663
Z1226 serW infA
Z1664 serX ycdW
Insertion sequences (IS) or IS-associated genes
Transposase-encoding genes
B.
clpA serW infA
ycdU serX ycdW
125
126

Fig. S1. PCR strategy to characterize tellurite resistance (Tel^{R})-encoding islands and their integration sites in L and S colonies of *E. coli* O157:H7 strains.

A. Physical map of O island (OI) 48 and its flanks in *E. coli* O157:H7 strain EDL933 and positions of primers used to map Tel^{R}-encoding islands. Large arrows indicate ORFs of OI 48 (designations above arrows). Striped arrows denote putative transposases. Dotted arrows indicate insertion sequences (IS) and IS-associated genes. *ter* genes are grey. The duplicated arrows at the 5´ and 3´ends of the island indicate respective ORFs at these positions in OI 48 (Z1559 [putative P4-family integrase of OI 48] and Z1664, respectively; lower arrows) and in OI 43 (Z1120 [putative P4-family integrase of OI 43] and Z1226, respectively; upper arrows). Moreover, genes upstream of the first ORF of OI 48 (*ycdU*) and OI 43 (*clpA*) and downstream of the last ORF of OI 48 (*serX*, *ycdW*) and OI 43 (*serW*, *infA*) are indicated by double arrows. The scale (in bp) is above the graph. Small arrows indicate positions of PCR primers; the numbers below the small arrows indicate PCR numbers in Table S1, which describes PCR conditions.

B. Strategy to investigate occupancy of *serW* and *serX*, the respective integration sites for OI 43 and OI 48 in EDL933.

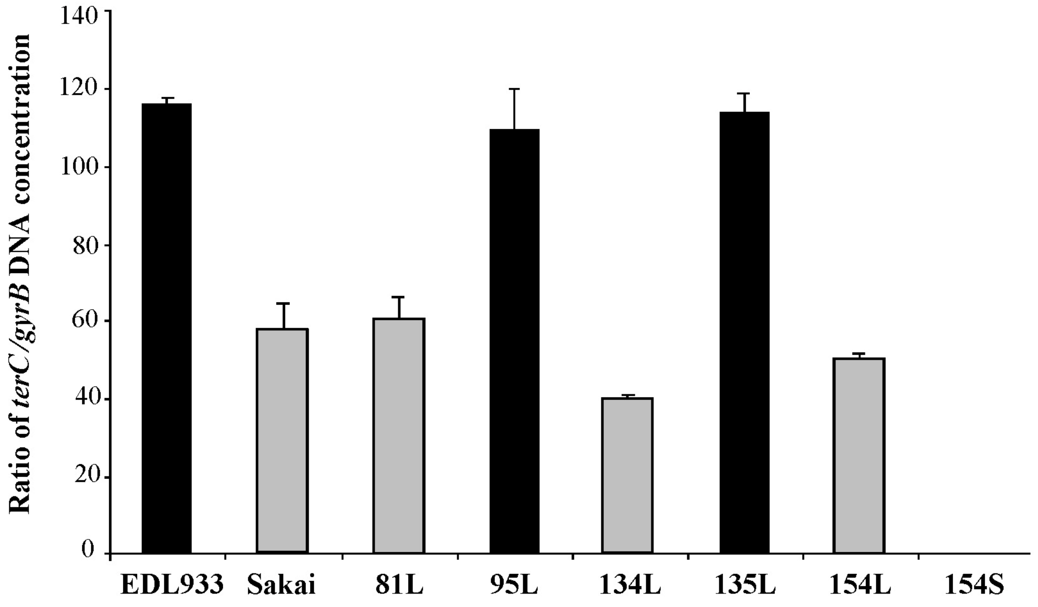

Fig. S2. Number of copies of Tel^R-encoding island in L strains. The *terC* DNA was quantified using light-cycler PCR and normalized to *gyrB* DNA. The *terC*/*gyrB* DNA ratio for each strain represents a mean ± standard deviation from three independent experiments. Strains with two copies or one copy of *terC* are depicted by black or grey bars, respectively. Strains EDL933 (GenBank accession no. AE005174; 2 copies of the *ter* cluster), Sakai RIMD 0509952 (GenBank accession no. NC_002695; 1 copy of the *ter* cluster), and 154S (lacking the *ter* cluster) were used as controls.

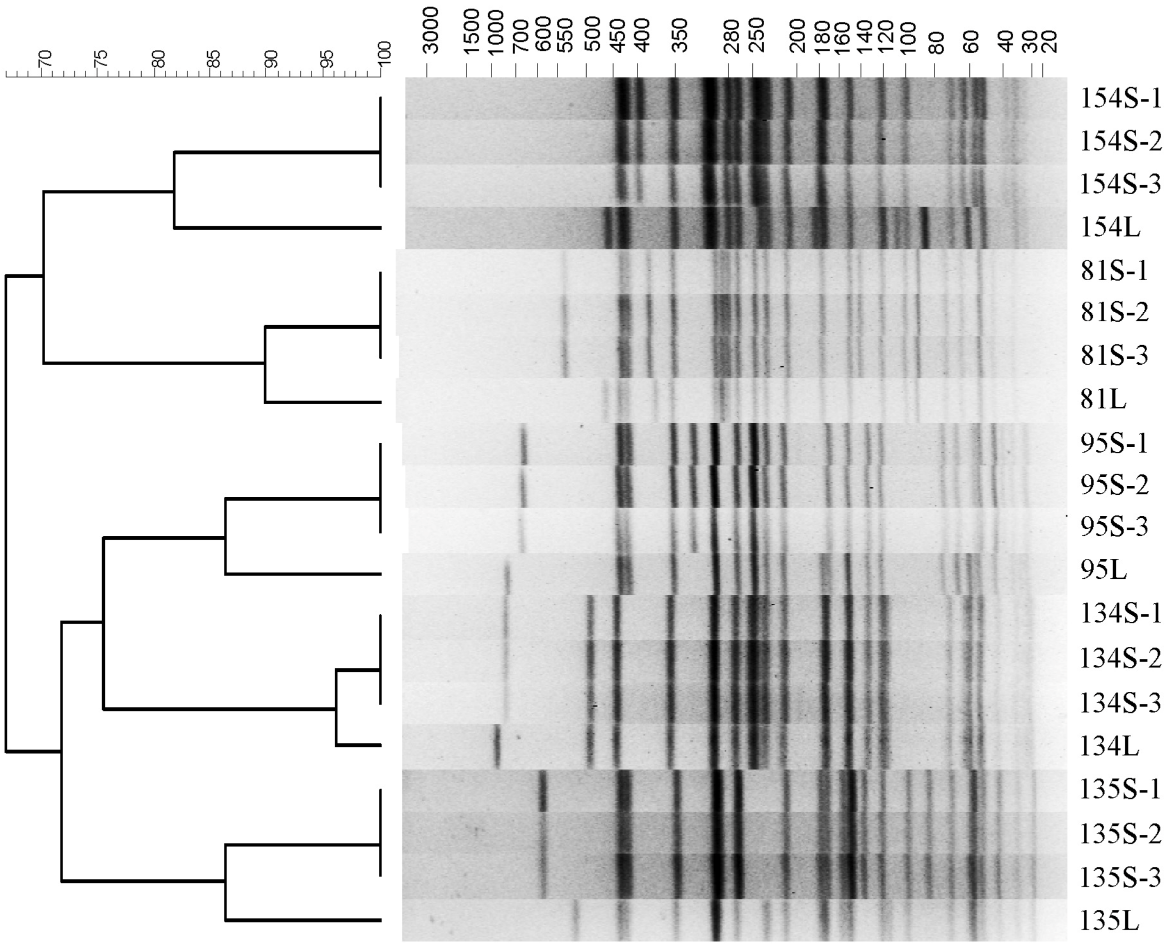

Fig. S3. PFGE of *Xba*I-digested genomic DNA from L and S strains. Chromosomal DNA from strains 81L, 95L, 134L, 135L, and 154 L and from three independent S colonies derived from each L strain was digested with *Xba*I and subjected to PFGE as described in Experimental procedures. The cluster analysis was performed using UPGMA (Unweighted Pair Group Method with Arithmetic Mean) in BioNumerics software 5.1. Strain numbers are shown to the right from the gel (three independent S colonies derived from each L strain are designated by strain number and the end numbers 1 to 3). Molecular weight scale (based on *Xba*I-digested DNA of *Salmonella enterica* serovar Braenderup strain H9812) is given above the gel.

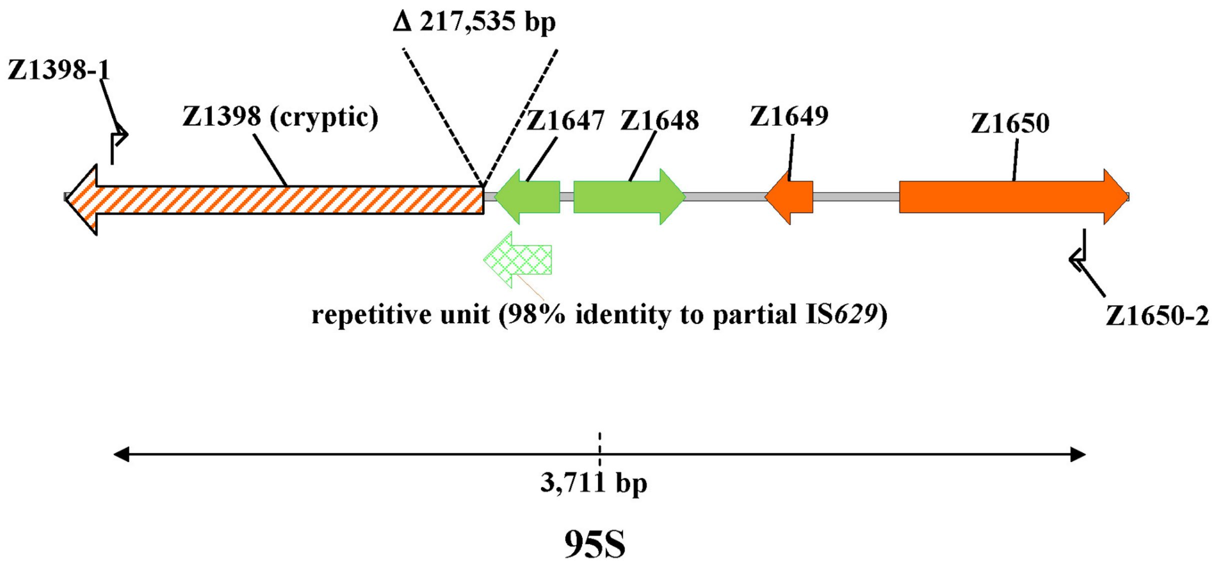

Fig. S4. Deletion of OI 48 and the upstream core genome region in strain 95S. The size and genetic organisation of the connecting fragment amplified with primers Z1398-1 and Z1650-2 (small arrows) that spans the internal deletion of OI 48 plus deleted parts of the core chromosome. Position and size of the deleted DNA region (217,535 bp) is indicated above the graph by dotted lines. ORFs with similarity to mobile genetic elements (putative transposases and insertion sequences) are highlighted in green.

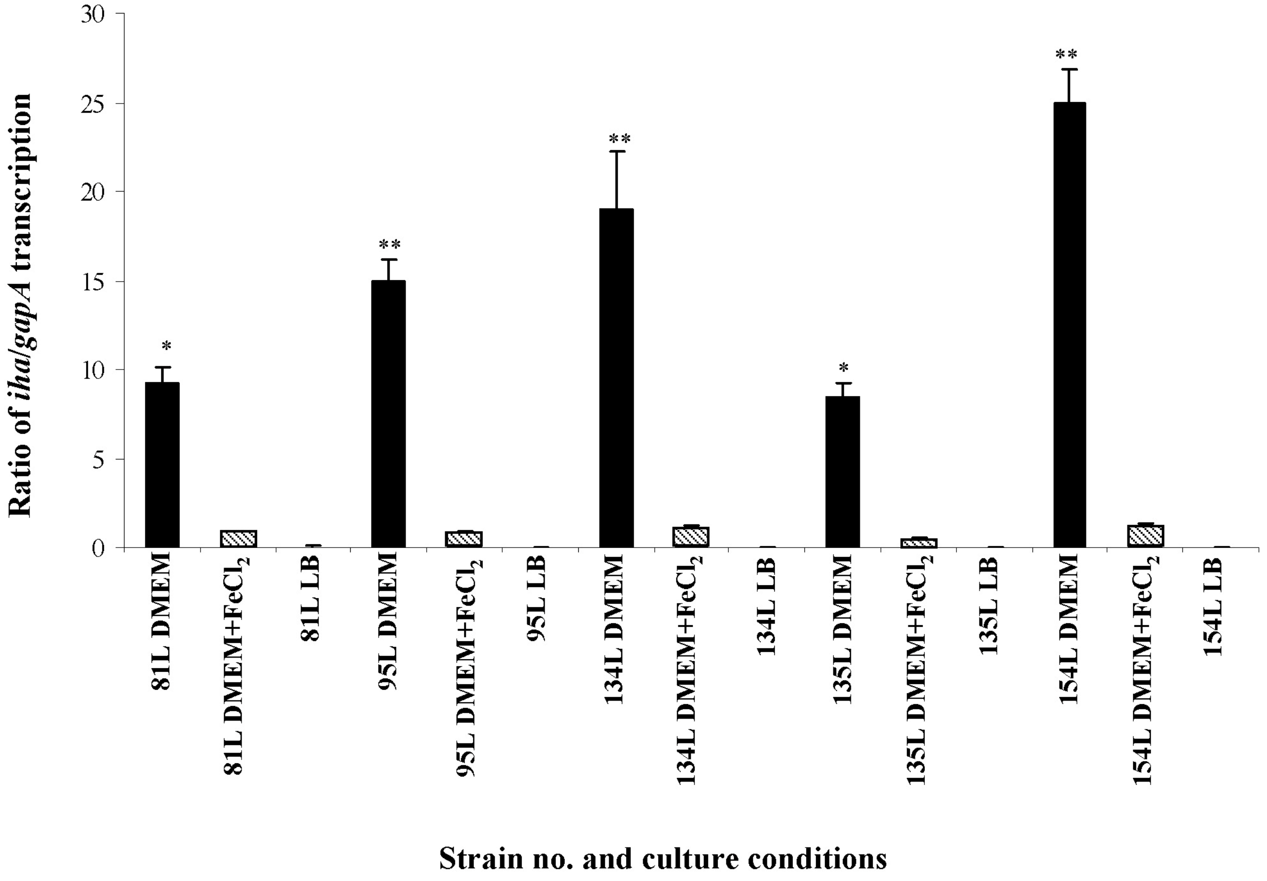

Fig. S5. *iha* transcription in L strains is upregulated in iron-limited conditions. The *iha*-positive L strains were cultured in DMEM (iron content < 0.05 µg ml^{-1}), DMEM with 10 µM $FeCl_2$ (iron content 0.50 µg ml^{-1}) or LB broth (iron content 0.59 µg ml^{-1}) and *iha* transcription was determined using quantitative real-time reverse transcription PCR and normalized to *gapA* transcription. The ratio of *iha*/*gapA* transcription was 8.4-24.9, ≤ 1.0, and ≤ 0.04, respectively. Data are presented as means ± standard deviations from three experiments performed with three independently isolated RNA preparations. Differences between relative transcription of *iha* in DMEM and in each of the other two media are indicated * ($p < 0.05$) and ** ($p < 0.001$) (unpaired Student´s *t* test).

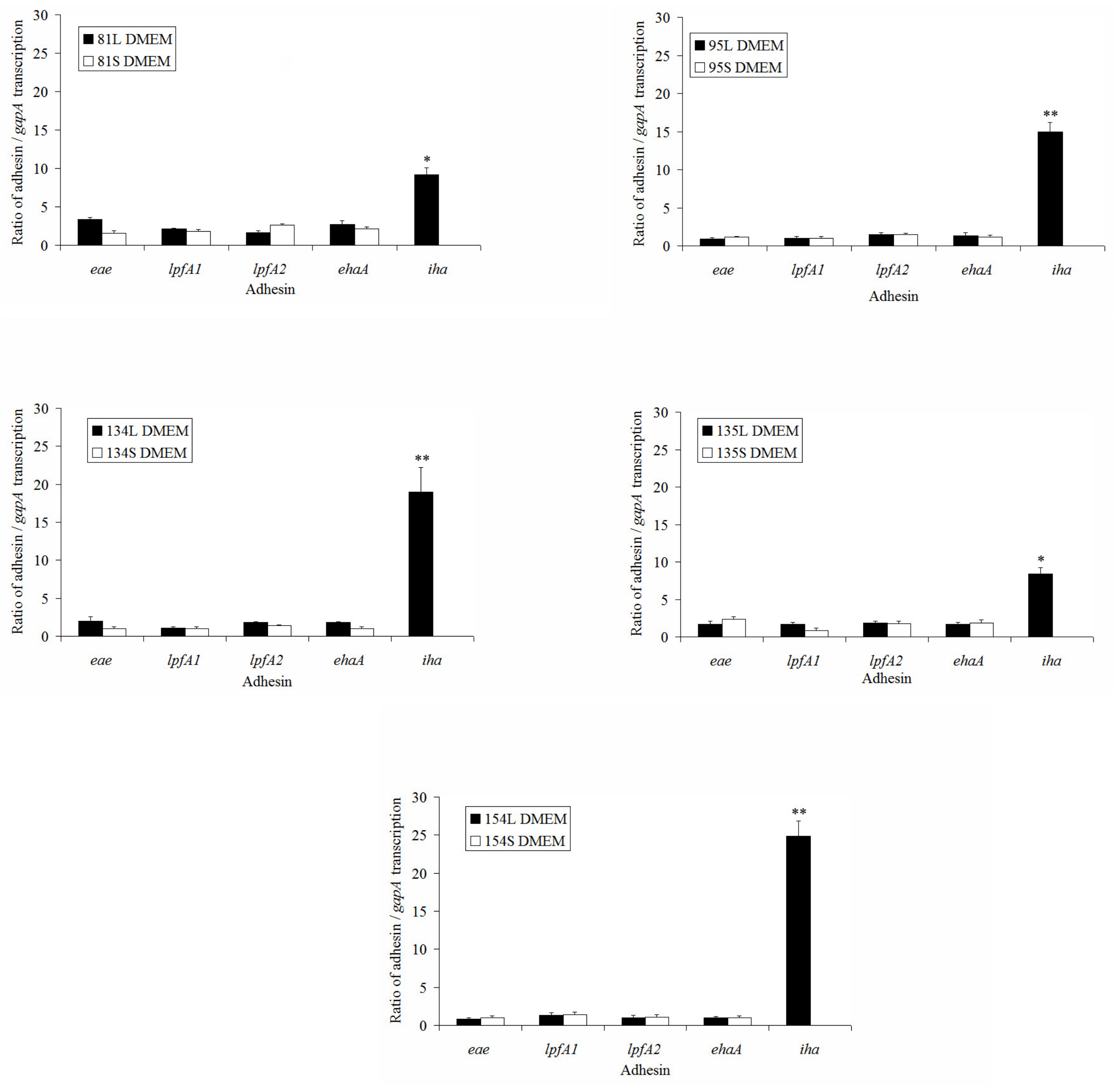

Fig. S6. Transcription analysis of *iha* and non-*iha* adhesin genes (*eae*, *lpfA1*, *lpfA2*, *ehaA*) in L and S strains grown under iron-limited conditions. The strains were cultured in DMEM (iron content < 0.05 µg ml^{-1}) and transcription of each gene was determined using quantitative real-time reverse transcription PCR and normalized to *gapA* transcription. Data are presented as means ± standard deviations from three experiments performed with three independently isolated RNA preparations. Differences between relative transcription of *iha* and each of the non-*iha* adhesin genes are indicated * ($p < 0.05$) and ** ($p < 0.001$) (unpaired Student´s *t* test).

Table S1. PCR primers and conditions used for mapping and analysis of deletions of Tel^{R}-encoding islands.

PCR No.	Primer[a]	Sequence (5′ - 3′)	Target	PCR conditions[b]			PCR product (bp)	Position of the primer[c]
				Denaturing	Annealing	Extension		
1	clpA-f<br>int-4	CTGTTTGGTTCGCTGGTGGAC<br>CCATTTCCCCCGCCCCTTTAC	*clpA*-integrase OI 43 (left junction of OI 43 and the core genome)	94°C, 30 s	56°C, 60 s	72°C, 60 s	756	2161-2181[d]<br>280-260
2	serX-F<br>OI 48 CI-R	TGTCGATTCCTCTGGCATAA<br>AAGTTGCTCTCGCATTATCC	*ycdU*-integrase OI 48 (left junction of OI 48 and the core genome)	95°C, 45 s	58°C, 45 s	72°C, 90 s	678	913-932[e]<br>397-378
3	int-1<br>int-2	AATCTGAGAAAGTCGCTATCC<br>CACTGACGGCTTTCTTTTTAC	integrase OI 43/OI 48	94°C, 30 s	54°C, 60 s	72°C, 60 s	708	435-455<br>1142-1122
4	Z1559-1<br>Z1560-2	GAATGAAAGCCAGGATAC<br>ATAAGCCTGTTTGATGGA	Z1559-Z1560	94°C, 30 s	50°C, 60 s	72°C, 60 s	767	972-989<br>1738-1721
5	Z1560-1<br>Z1563-2	TAGCCAGAACACCAAAAC<br>AGTTCAGGCATCCGTAAG	Z1560-Z1563	94°C, 30 s	51°C, 60 s	72°C, 90 s	1,228	3310-3327<br>4537-4520
6	Z1563-1<br>Z1565-4	CAGCAGTCAAAGGTGTTA<br>CGGATAAGGATTTTCTGG	Z1563-Z1565	94°C, 30 s	49°C, 60 s	72°C, 60 s	743	4708-4725<br>5450-5433
7	Z1565-1<br>Z1565-2	AACGCATCTGAATAAACC<br>AGACAACCGCACATACTC	Z1565	94°C, 30 s	51°C, 60 s	72°C, 60 s	688	5849-5866<br>6536-6518
8	Z1565-3<br>Z1566-2	TGTCGCACCTAACTTTGA<br>ATTTTTTCATACCTATAA	Z1565-1566	94°C, 30 s	48°C, 60 s	72°C, 60 s	381	6779-6796<br>7159-7142

PCR No.	Primer[a]	Sequence (5´ - 3´)	Target	PCR conditions[b]			PCR product (bp)	Position of the primer[c]
				Denaturing	Annealing	Extension		
9	Z1567-1<br>Z1567-2	ATTCATTTTATCGGCTAC<br>TACGCCTCAGAATAATAC	Z1567	94°C, 30 s	49°C, 60 s	72°C, 60 s	256	7885-7902<br>8140-8123
10	Z1568-1<br>Z1568-4	ATCAGTGGGCTTTTCTTG<br>ATCGCAGAAAATGAAACC	Z1568	94°C, 30 s	53°C, 60 s	72°C, 60 s	862	9120-9137<br>9981-9964
11	Z1565-3<br>Z1568-2	TGTCGCACCTAACTTTGA<br>GAGACCAGACGGGCATTT	Z1565-Z1568	94°C, 30 s	51°C, 60 s	72°C, 120 s	1,628	6779-6796<br>8406-8389
12	Z1569-1<br>Z1569-2	TTTGCCATAATCGCTTCT<br>ATCGGTAATCTTGAGGAA	Z1569	94°C, 30 s	50°C, 60 s	72°C, 60 s	212	10907-10924<br>11118-11101
13	Z1568-3<br>Z1570-2	CGTATCCTGTGCTGCTCA<br>TCCTGTTCTTCGGCTCTG	Z1568-Z1570	94°C, 60 s	54°C, 60 s	72°C, 90 s	1,042	10605-10622<br>11646-11629
14	Z1571-1<br>Z1571-2	TACCTTCCAGCAGCATCG<br>GTTACCTTTCGGGACCAA	Z1571	94°C, 30 s	56°C, 60 s	72°C, 60 s	290	13097-13114<br>13386-13369
15	Z1572-1<br>Z1572-2	GCTTACGATTACGCTGAA<br>TATGGAAGAACGGAACAG	Z1572	94°C, 30 s	52°C, 60 s	72°C, 60 s	255	13858-13875<br>14112-14095
16	Z1573-1<br>Z1573-4	GTATCCAGGAGCGGTTAT<br>GAGATATTCCCCAGACGA	Z1573	94°C, 60 s	54°C, 60 s	72°C, 60 s	758	14316-14332<br>15073-15056
17	Z1570-1<br>Z1573-2	CAGCAGGAAGATGTCGTC<br>CCGCTCATCACTTTGTTT	Z1570-Z1573	94°C, 60 s	54°C, 60 s	72°C, 120 s	1,423	12962-12979<br>14384-14367
18	Z1574-3<br>Z1574-4	CAAAGCAGACAAAATGGA<br>GGTATTTATGCTGGTTGC	Z1574	94°C, 60 s	53°C, 60 s	72°C, 60 s	332	15333-15350<br>15664-15647

PCR No.	Primer[a]	Sequence (5′ - 3′)	Target	PCR conditions[b]			PCR product (bp)	Position of the primer[c]
				Denaturing	Annealing	Extension		
19	Z1574-1<br>Z1575-2	CTCTCGGTGTTGGACTGG<br>CAAACCGAACGCTGAAGA	Z1574-Z1575	94°C, 30 s	53°C, 60 s	72°C, 60 s	462	15458-15475<br>15919-15902
20	Z1575-1<br>Z1575-4	TATTATTTTCCCTGTCAC<br>AGGAGGTTCTGTTATGGA	Z1575	94°C, 60 s	48°C, 60 s	72°C, 60 s	244	15837-15854<br>16080-16063
21	Z1576-3<br>Z1576-4	CGGAGACGCCATTATTTT<br>TTTGAACTCCTGATGTCG	Z1576	94°C, 30 s	48°C, 60 s	72°C, 60 s	202	16141-16158<br>16342-16325
22	Z1576-3<br>Z1577-2	CGGAGACGCCATTATTTT<br>ATTGATGACCTCTTTGCT	Z1576-Z1577	94°C, 30 s	50°C, 60 s	72°C, 60 s	439	16141-16158<br>16579-16562
23	Z1577-1<br>Z1578-2	AGGTCATCAATCTGGTAT<br>GCAACTAAAATCGTAATC	Z1577-Z1578	94°C, 30 s	48°C, 60 s	72°C, 60 s	824	16569-16586<br>17392-17375
24	Z1578-3<br>Z1578-2	GAGTGCATTCTGGTTCTG<br>GCAACTAAAATCGTAATC	Z1578	94°C, 30 s	47°C, 60 s	72°C, 60 s	224	17169-17186<br>17392-17375
25	Z1576-1<br>Z1579-4	CGGCATCATTCATACTGG<br>TCGTTACTGGGGTTATGG	Z1576-Z1579	94°C, 60 s	51°C, 60 s	72°C, 120 s	1,243	16351-16368<br>17593-17576
26	Z1579-1<br>Z1579-2	GGATTCGCTTACATACAC<br>TGTGGTTTTTATTTACGG	Z1579	94°C, 30 s	48°C, 60 s	72°C, 60 s	630	18145-18162<br>18774-18757
27	Z1580-1<br>UreD-r	TGTCGCTTCCCCAAGAAAAGG<br>GCGTGGCTCCGGCGTAGTTTT	Z1580-*ureD* (Z1581)	94°C, 30 s	58°C, 60 s	72°C, 90 s	1,081	19578-19598<br>20658-20638
28	UreD-f<br>UreA-r	CGTCATCATGTCGGTCTGCTCA<br>CAGATTATCGGATTATGGACGGTA	*ureD-ureA*<br>(Z1581-Z1582)	94°C, 30 s	58°C, 60 s	72°C, 90 s	1,040	20070-20091<br>21109-21086

PCR No.	Primer[a]	Sequence (5´ - 3´)	Target	PCR conditions[b]			PCR product (bp)	Position of the primer[c]
				Denaturing	Annealing	Extension		
29	UreA-f<br>UreB-r	GACTCCAAGAGAAAAAGACAAACTA<br>ACCCATTATATCTCCGCGGAAACCG	*ureA-ureB*<br>(Z1582-Z1583)	94°C, 30 s	56°C, 60 s	72°C, 60 s	600	20816-20840<br>21415-21391
30	UreB-f<br>UreC-r	GCTACCTGCAGTATTATCGTTGAA<br>GAGGAAGGCAGAATATTGGG	*ureB-ureC*<br>(Z1583-Z1584)	94°C, 30 s	57°C, 60 s	72°C, 90 s	1,146	21176-21199<br>22321-22302
31	UreC-f<br>UreE-r	TCTAACGCCACAACCTGTAC<br>CGTGATTATGGGCGTGCGACTT	*ureC-ureE*<br>(Z1584-Z1585)	94°C, 30 s	56°C, 60 s	72°C, 120 s	1,666	21924-21943<br>23589-23568
32	UreE-f<br>UreF-r	GAGACCCCGGCTCAGACAACT<br>ACGGAATAATCGGGAATACTGGG	*ureE-ureF*<br>(Z1585-Z1586)	94°C, 30 s	60°C, 60 s	72°C, 90 s	1,110	23169-23189<br>24278-24256
33	UreF-f<br>UreG-r	ACTGGAGTGGGCAGTGGAAGC<br>GATGTTTTGCAGACCTTCACCAC	*ureF-ureG*<br>(Z1586-Z1587)	94°C, 30 s	59°C, 60 s	72°C, 90 s	1,182	23693-23713<br>24874-24852
34	UreG-f<br>Z1588-2	GGTCCGGTCGGCTCAGGTAAA<br>GTTTGAAAAATCGGCATCTGG	*ureG* (Z1587)-Z1588	94°C, 30 s	57°C, 60 s	72°C, 60 s	699	24335-24355<br>25033-25013
35	Z1589-3<br>Z1589-2	AACTGATAACCGATACCG<br>AGGCACTTTAGGACAATG	Z1589	94°C, 30 s	51°C, 60 s	72°C, 60 s	226	25172-25189<br>25397-25380
36	Z1590-1<br>Z1590-2	CATCTGGGTTTTTACTTC<br>AAAAGTTATGATGATTGC	Z1590	94°C, 30 s	47°C, 60 s	72°C, 40 s	106	25584-25601<br>25689-25672
37	Z1591-1<br>Z1591-4	ATTGAACTGGATGGTGTA<br>CCTTTTTTCGCATCAACA	Z1591	94°C, 30 s	48°C, 60 s	72°C, 40 s	155	26227-26244<br>26381-26364
38	Z1589-1<br>Z1591-2	GAGTACCGAGCCCTTTTC<br>AAACGGATGCGACTTAGA	Z1589-Z1591	94°C, 30 s	52°C, 60 s	72°C, 90 s	945	25348-25365<br>26292-26275

PCR No.	Primer[a]	Sequence (5´ - 3´)	Target	PCR conditions[b]			PCR product (bp)	Position of the primer[c]
				Denaturing	Annealing	Extension		
39	Z1592-3<br>Z1592-4	GCCTGACCATGTTGCTGA<br>GCAAGAACGCCGCAATTT	Z1592	94°C, 30 s	52°C, 60 s	72°C, 40 s	93	26724-26741<br>26816-26799
40	Z1593-1<br>Z1593-2	ACTTTATTGATTTACTGC<br>ATAGTTTTAGCAATACCT	Z1593	94°C, 30 s	45°C, 60 s	72°C, 40 s	135	27259-27276<br>27393-27376
41	Z1594-1<br>Z1594-4	ATAGTGGATTTCGTTTCA<br>CCCAGAAACACGATAAAG	Z1594	94°C, 30 s	47°C, 60 s	72°C, 60 s	622	27779-27796<br>28400-28383
42	Z1592-1<br>Z1594-2	TGTAAACCGTAAGAAACC<br>AATAATGAAACGAAATCC	Z1592-Z1594	94°C, 30 s	49°C, 60 s	72°C, 90 s	983	26819-26836<br>27801-27784
43	Z1595-3<br>Z1595-4	GCAGACTGGCATAAAAAC<br>ACACGCAAGCGACCAAAT	Z1595	94°C, 30 s	48°C, 60 s	72°C, 40 s	121	28839-28856<br>28959-28942
44	Z1596-1<br>Z1596-2	TTTCGGGCGCATCATCAG<br>GCCGGAACAATACCTCAG	Z1596	94°C, 30 s	54°C, 60 s	72°C, 40 s	131	29429-29446<br>29559-29542
45	Z1597-1<br>Z1597-4	CTCAAGGGGCAGCAACTC<br>ATACGGCAATGGCGACAA	Z1597	94°C, 30 s	57°C, 60 s	72°C, 60 s	396	29928-29945<br>30323-30306
46	Z1595-1<br>Z1597-2	CGCTTCTTATGACTGTGC<br>GAAGACCGACTGGAAGAG	Z1595-Z1597	94°C, 30 s	54°C, 60 s	72°C, 90 s	1,059	28902-28919<br>29960-29943
47	Z1598-3<br>Z1598-4	TAAACTGGCTTCACAACC<br>GGCGGAACTCAACTTTAC	Z1598	94°C, 30 s	52°C, 60 s	72°C, 60 s	278	30780-30797<br>31057-31040
48	Z1599-1<br>Z1599-2	TATGCGTAAATCCTTCAA<br>TGGATACCTTACCGTCAC	Z1599	94°C, 30 s	52°C, 60 s	72°C, 60 s	209	31187-31204<br>31395-31378

PCR No.	Primer[a]	Sequence (5´ - 3´)	Target	PCR conditions[b]			PCR product (bp)	Position of the primer[c]
				Denaturing	Annealing	Extension		
49	Z1600-3<br>Z1600-4	CGGGCATTACTCTCCAAC<br>CGCTGCCAAAGAACATAA	Z1600	94°C, 30 s	56°C, 60 s	72°C, 60 s	745	32214-32231<br>32958-32941
50	Z1598-1<br>Z1600-2	TCCGCTTTTATCCACTCC<br>TATTTCCCGCTCCTCCTC	Z1598-Z1600	94°C, 30 s	55°C, 60 s	72°C, 60 s	696	30993-31010<br>31688-31671
51	Z1601-1<br>Z1601-2	ATGTGCTGCTGGCGATAC<br>TGGAAGAGCAGATGAAGC	Z1601	94°C, 30 s	55°C, 60 s	72°C, 60 s	496	33376-33393<br>33871-33854
52	Z1602-1<br>Z1602-4	ATGCCCTTTCACCTGTTG<br>ATGCTGACGCCTGATAAC	Z1602	94°C, 30 s	54°C, 60 s	72°C, 60 s	315	34098-34115<br>34412-34395
53	Z1600-1<br>Z1602-2	AGGCGGATAATAATGCTG<br>TTCACTGGCTGGTCTTTG	Z1600-Z1602	94°C, 30 s	55°C, 60 s	72°C, 120 s	1,264	32881-32898<br>34144-34127
54	TerW-1<br>TerW-2	TTCTCTACCGCTTCACTT<br>TCAAATACAGCAAGGCAG	*terW* (Z1603)	94°C, 60 s	55°C, 60 s	72°C, 60 s	250	35047-35064<br>35296-35279
55	Z1604-3<br>Z1604-4	GTGGTGGGCATAGTTTTC<br>CGAGAGCGAAGGCATTAG	Z1604	94°C, 30 s	54°C, 60 s	72°C, 60 s	490	35753-35770<br>36242-36225
56	Z1605-3<br>Z1605-4	GCGGTCAGGGCAAAATCA<br>ATCCCCGTCGCCCTCAAA	Z1605	94°C, 30 s	58°C, 60 s	72°C, 60 s	476	37024-37041<br>37499-37482
57	Z1604-1<br>Z1605-2	GGTATTCTCACGCTCCAC<br>GGGACCTATGCCAATCTG	Z1604-Z1605	94°C, 30 s	55°C, 60 s	72°C, 60 s	444	36119-36136<br>36562-36545
58	Z1606-1<br>Z1606-2	ACGGTATCGGCAAGCATC<br>TGACATGGGGAAAACCTC	Z1606	94°C, 30 s	56°C, 60 s	72°C, 60 s	402	37986-38003<br>38387-38370

PCR No.	Primer[a]	Sequence (5′ - 3′)	Target	PCR conditions[b]			PCR product (bp)	Position of the primer[c]
				Denaturing	Annealing	Extension		
59	Z1607-3<br>Z1607-4	GACTGCCAGCCCTTTCTC<br>ATCGCACCCTGAATCCAC	Z1607	94°C, 30 s	56°C, 60 s	72°C, 60 s	500	38898-38915<br>39397-39380
60	Z1605-1<br>Z1607-2	CTCCAGGCAAATCACCAG<br>GCTGGCTGCTTGAAAAAC	Z1605-Z1607	94°C, 30 s	56°C, 60 s	72°C, 120 s	1,376	37521-37538<br>38896-38879
61	Z1608-1<br>Z1608-2	CCCCTTGTGGACGGAGAC<br>TACTCGGCAGGCACATTC	Z1608	94°C, 30 s	57°C, 60 s	72°C, 60 s	668	39817-39834<br>40484-40467
62	Z1609-3<br>Z1609-4	GCTTCCTTCGTTTACCAG<br>AACCATTCAGACCTACGG	Z1609	94°C, 30 s	54°C, 60 s	72°C, 60 s	550	40954-40971<br>41503-41486
63	Z1607-1<br>Z1609-2	AAAGGAAATGGCGGACTC<br>CCTTCCTGGGTTATTTGC	Z1607-Z1609	94°C, 30 s	56°C, 60 s	72°C, 90 s	1,135	39664-39681<br>40798-40781
64	Z1609-1<br>Z1610-2	GTGGATGCCGTAGGTCTG<br>ACCGAGACCGAAATGAAG	Z1609-Z1610	94°C, 30 s	50°C, 60 s	72°C, 60 s	631	41479-41496<br>42109-42092
65	TerZ1<br>TerA2	GACGGTATCACTCAGCAAAGAATC<br>TGGCGGGTCAGTTCGTCAC	*terZ-terB*<br>(Z1610-Z1612)	94°C, 30 s	58°C, 60 s	72°C, 150 s	1,800	42052-42075<br>43851-43833
66	TerA1<br>TerB2	TATCGTTTCAGCGGTTATTC<br>TCGCAACGGCAATACCAACACG	*terZ-terB*<br>(Z1610-Z1612)	94°C, 30 s	56°C, 60 s	72°C, 120 s	1,568	42586-42605<br>44153-44132
67	TerB1<br>TerC2	AGGCCGTGACGAACTGACC<br>GAAACACTCATAAAATAACCTCTT	*terB-terD*<br>(Z1612-Z1614)	94°C, 30 s	55°C, 60 s	72°C, 120 s	1,540	43828-43846<br>45367-45344
68	TerC1<br>TerD2	TCCTGGCGCTGAAAGAT<br>CCGAACAGCATGGCAGTCT	*terB-terD*<br>(Z1612-Z1614)	94°C, 30 s	56°C, 60 s	72°C, 150 s	1,742	44088-44104<br>45829-45811

PCR No.	Primer[a]	Sequence (5′ - 3′)	Target	PCR conditions[b]			PCR product (bp)	Position of the primer[c]
				Denaturing	Annealing	Extension		
69	TerD1<br>TerE2	AGTAAAGCAGCTCCGTCAAT<br>CCGTCCCGTTGTCGTTGTTGTAA	*terD-terE*<br>(Z1614-Z1615)	94°C, 30 s	55°C, 60 s	72°C, 90 s	1,023	45396-45415<br>46418-46396
70	TerE1<br>TerF2	TAAAAGGCGGCAACGTATCTCTGA<br>CAATGACAACGGTGATCG	*terE-terF*<br>(Z1615-Z1616)	94°C, 30 s	56°C, 60 s	72°C, 120 s	1,267	46020-46043<br>47286-47269
71	Z1616-1<br>Z1617-2	GCATTGAGATAGCACTGG<br>GTGAATGCGAAACTGAAC	Z1616-Z1617	94°C, 30 s	53°C, 60 s	72°C, 90 s	1,058	47221-47238<br>48278-48261
72	Iha-I<br>Iha-II	CAGTTCAGTTTCGCATTCACC<br>GTATGGCTCTGATGCGATG	*iha* (Z1617)	94°C, 30 s	56°C, 60 s	72°C, 90 s	1,305	48259-48279<br>49563-49545
73	Z1617-1<br>Z1619-2	GACAGGGAATGACTACGG<br>TGTAAAGCGGGGAGTAAC	Z1617-Z1619	94°C, 30 s	51°C, 60 s	72°C, 60 s	785	49952-49969<br>50736-50719
74	Z1619-1<br>Z1620-2	AAAACTCTTGACGCATAC<br>TAGCCATACGACTTTACC	Z1619-Z1620	94°C, 30 s	48°C, 60 s	72°C, 60 s	764	50840-50857<br>51603-51586
75	Z1620-3<br>Z1622-2	AAAGTCGTATGGCTATGG<br>ACATTCCGTCCAACAGAG	Z1620-Z1622	94°C, 30 s	49°C, 60 s	72°C, 60 s	841	51589-51606<br>52429-52412
76	Z1623-1<br>Z1623-2	AGATGGATTTCGCTGGAC<br>ATGACATTGTAATAAGGC	Z1623	94°C, 30 s	46°C, 60 s	72°C, 40 s	112	53166-53183<br>53277-53260
77	Z1624-1<br>Z1624-2	AGTGGAAAATAGGTTAGG<br>AAAATAAAAAGGCTGGAG	Z1624	94°C, 30 s	48°C, 60 s	72°C, 40 s	104	53491-53508<br>53594-53577
78	Z1622-1<br>Z1625-2	ACACAGGAGCCAGAATAC<br>TAAATCAGGCAAAACCAG	Z1622-Z1625	94°C, 30 s	51°C, 60 s	72°C, 120 s	1,438	52528-52545<br>53965-53948

PCR No.	Primer[a]	Sequence (5´ - 3´)	Target	PCR conditions[b]			PCR product (bp)	Position of the primer[c]
				Denaturing	Annealing	Extension		
79	Z1625-1<br>Z1626-2	AGACTCTCGTTGCTGGTG<br>CGGGATACCAGGGATTAC	Z1625-Z1626	94°C, 30 s	53°C, 60 s	72°C, 60 s	639	54043-54060<br>54681-54664
80	Z1627-1<br>Z1627-2	ACAACAAGCCACGACAGC<br>TGCTCCACTTCACTTTTG	Z1627	94°C, 30 s	51°C, 60 s	72°C, 40 s	179	54773-54790<br>54951-54934
81	Z1628-1<br>Z1628-2	GCGCTGGTATTGTGGAAC<br>TCAGGAAGGGATAACAAC	Z1628	94°C, 30 s	51°C, 60 s	72°C, 40 s	105	55060-55077<br>55164-55147
82	Z1629-1<br>Z1629-2	TACCCATTGATTTCTCTG<br>TATATTTAGCAGCAACCA	Z1629	94°C, 30 s	49°C, 60 s	72°C, 60 s	313	55739-55756<br>56051-56034
83	Z1626-1<br>Z1630-2	ATCCCTGGTATCCCGTAA<br>TTTCCTTTACAGCACCTC	Z1626-Z1630	94°C, 30 s	51°C, 60 s	72°C, 120 s	1,612	54667-54684<br>56278-56261
84	Z1630-1<br>Z1631-2	GATTGTGTTTGGCGAAGG<br>CGCCTCCGAAAGATGTAA	Z1630-Z1631	94°C, 30 s	52°C, 60 s	72°C, 60 s	603	56574-56591<br>57176-57159
85	Z1632-1<br>Z1632-2	AGATAATGCCCGATGACC<br>CGGGCAGTGATGTGATTG	Z1632	94°C, 30 s	55°C, 60 s	72°C, 60 s	385	57255-57272<br>57639-57622
86	Z1631-1<br>Z1633-2	TAAAGAACCGACTCCAAG<br>TTGTTTTCCTCATTGTTC	Z1631-Z1633	94°C, 30 s	51°C, 60 s	72°C, 90 s	1,094	57133-57150<br>58226-58209
87	Z1634-1<br>Z1634-2	GTTGGCATTTCACTTCAC<br>CTGCGACATAAAGGACTG	Z1634	94°C, 30 s	53°C, 60 s	72°C, 60 s	403	59167-59184<br>59569-59552
88	Z1633-1<br>Z1635-2	AAGGTGGAAGTGCTGAAA<br>TCGTTCCGTTCTGTAAAA	Z1633-Z1635	94°C, 30 s	51°C, 60 s	72°C, 90 s	1,166	58572-58589<br>59737-59720

PCR No.	Primer[a]	Sequence (5′ - 3′)	Target	PCR conditions[b]			PCR product (bp)	Position of the primer[c]
				Denaturing	Annealing	Extension		
89	Z1635-1<br>Z1636-2	TGTTCAGGTTTGGCAGTC<br>ACAAGCCCAGAAAACCAC	Z1635-Z1636	94°C, 30 s	54°C, 60 s	72°C, 60 s	450	60166-60183<br>60615-60598
90	Z1637-1<br>Z1637-2	TTTATTCTGTTTCTTCCC<br>TTACCGAAAGTCAATCAG	Z1637	94°C, 30 s	50°C, 60 s	72°C, 40 s	175	60686-60703<br>60860-60843
91	Z1638-3<br>Z1638-4	AAAATCAGCCACCCACAG<br>GCAACAGCGACATCATCC	Z1638	94°C, 30 s	56°C, 60 s	72°C, 60 s	316	61952-61969<br>62267-62250
92	Z1636-1<br>Z1638-2	GTTTTCTGGGCTTGTCTC<br>GGCAGAAGCAGAAAAAGC	Z1636-Z1638	94°C, 30 s	51°C, 60 s	72°C, 90 s	929	60601-60618<br>61529-61512
93	Z1639-1<br>Z1639-2	AACTCCGCCTTCGCAAAA<br>CGTTTTTCCCCCGAGGTC	Z1639	94°C, 30 s	57°C, 60 s	72°C, 60 s	290	62381-62398<br>62670-62653
94	Z1640-1<br>Z1640-2	GCTGATGCGTTACCACAC<br>ACGATTTGACAGGCTTTG	Z1640	94°C, 30 s	54°C, 60 s	72°C, 60 s	504	63088-63105<br>63591-63574
95	Z1638-1<br>Z1640-4	ATGATGTCGCTGTTGCTG<br>TGCTTCATCTCCGCTACG	Z1638-Z1640	94°C, 30 s	55°C, 60 s	72°C, 60 s	680	62252-62269<br>62931-62914
96	Z1641-1<br>Z1641-2	GAAATGACGACAGAGAGG<br>CGTTTTTACTCTGTCCTG	Z1641	94°C, 30 s	50°C, 60 s	72°C, 60 s	429	64220-64237<br>64648-64631
97	Z1642-3<br>Z1642-4	GAAAAGATGGACAAACAG<br>TGTTTCTGAATCCAATCC	Z1642	94°C, 30 s	49°C, 60 s	72°C, 60 s	370	64863-64880<br>65232-65215
98	Z1640-3<br>Z1642-2	GTTTGTGGCTGGTGTCTG<br>TGTGTTTTACCTGTTCCA	Z1640-Z1642	94°C, 30 s	51°C, 60 s	72°C, 90 s	986	63786-63803<br>64771-64754

PCR No.	Primer[a]	Sequence (5´ - 3´)	Target	PCR conditions[b]			PCR product (bp)	Position of the primer[c]
				Denaturing	Annealing	Extension		
99	Z1643-1<br>Z1643-2	CTTTATGGGGCTGGTGTC<br>GCTCCCCAGGTCATTCTC	Z1643	94°C, 30 s	55°C, 60 s	72°C, 60 s	544	67869-67886<br>68412-68395
100	Z1642-1<br>Z1643-4	GGTTATTGAAGTGGATGG<br>CACAAGATGGAAAAGCAC	Z1642-Z1643	94°C, 30 s	50°C, 60 s	72°C, 60 s	821	66665-66682<br>67485-67468
101	Z1644-1<br>Z1644-2	GGTGAGAAGAAACTGCTG<br>CCGCTAATCTGTTTCAAT	Z1644	94°C, 30 s	53°C, 60 s	72°C, 60 s	368	68766-68783<br>69133-69116
102	Z1645-1<br>Z1645-2	GTCATCCATCAACCAGTG<br>TGTTATTCGCTGTTTCTG	Z1645	94°C, 30 s	50°C, 60 s	72°C, 60 s	371	69702-69719<br>70072-70055
103	Z1643-3<br>Z1645-4	GCGTATGAAAGAAAGGAG<br>TTCATCGCATAAAAGTTG	Z1643-Z1645	94°C, 30 s	53°C, 60 s	72°C, 90 s	1,091	68526-68543<br>69616-69599
104	Z1646-1<br>Z1646-2	GATGAAAGTGAATGGTGT<br>TTGACAAGACGACGTTAG	Z1646	94°C, 30 s	48°C, 60 s	72°C, 40 s	151	70248-70265<br>70398-70381
105	Z1647-3<br>Z1647-4	CCAGCCTTTCCAGCAATC<br>TACGGCGGTCAGTTTACG	Z1647	94°C, 30 s	51°C, 60 s	72°C, 60 s	169	70970-70987<br>71138-71121
106	Z1645-3<br>Z1647-2	ATTTTATGAGCCCGATGC<br>CGGCAGAAGCAGAAAAAG	Z1645-Z1647	94°C, 30 s	53°C, 60 s	72°C, 60 s	880	70078-70095<br>70957-70940
107	Z1648-1<br>Z1648-2	TACAGGACTGGCGAAAAG<br>TCTGAGTGGAAGTGGATA	Z1648	94°C, 30 s	51°C, 60 s	72°C, 60 s	300	71208-71225<br>71507-71490
108	Z1649-1<br>Z1649-2	GCCGCCTCATCATTATCC<br>ACGATGAAAACTATGACG	Z1649	94°C, 30 s	47°C, 60 s	72°C, 40 s	94	71966-71983<br>72059-72042

PCR No.	Primer[a]	Sequence (5´ - 3´)	Target	PCR conditions[b]			PCR product (bp)	Position of the primer[c]
				Denaturing	Annealing	Extension		
109	Z1650-1<br>Z1650-2	TCGCTTTCCCGTCACATC<br>TGCCCCCAGACAGATTCC	Z1650	94°C, 30 s	58°C, 60 s	72°C, 60 s	665	72494-72511<br>73158-73141
110	Z1647-1<br>Z1650-4	TAAACTGACCGCCGTATG<br>GTAAGGCAGGGAGGAGAG	Z1647-Z1650	94°C, 30 s	52°C, 60 s	72°C, 120 s	1,371	71123-71140<br>72493-72476
111	Z1651-1<br>Z1651-2	ACCACCACGAACCTCACC<br>GACGGACGAAGCCATAGC	Z1651<br>(AIDA-I)	94°C, 30 s	57°C, 60 s	72°C, 60 s	532	75211-75228<br>75742-75725
112	Z1650-3<br>Z1651-4	AAACAGATGCGGTATTCC<br>ACAGCAGCAAGAGACAGC	Z1650-Z1651	94°C, 30 s	55°C, 60 s	72°C, 60 s	837	72978-72995<br>73814-73797
113	Z1652-1<br>Z1654-4	TGGGAAAACTATGACGAG<br>AATGCTGGTCCCTGAAAC	Z1652-Z1654	94°C, 30 s	54°C, 60 s	72°C, 120 s	1,369	78684-78701<br>80052-80035
114	Z1653-1<br>Z1653-2	CTGGCTGACGGAGATGAC<br>GATGCTGCTCTTCACTGC	Z1653	94°C, 30 s	55°C, 60 s	72°C, 60 s	545	79261-79278<br>79805-79788
115	Z1654-1<br>Z1654-2	CGTGCTGAACTGGGTGTC<br>GCAATACCGCCTCTGACC	Z1654	94°C, 30 s	57°C, 60 s	72°C, 60 s	487	80440-80457<br>80926-80909
116	Z1654-3<br>Z1656-2	CGGAAGAGGCAGATTTGG<br>GAAGACACGGGGCTCCAG	Z1654-Z1656	94°C, 30 s	56°C, 60 s	72°C, 120 s	1,472	80945-80961<br>82416-82399
117	Z1657-2<br>Z1658-2	ACACCCAGTAAGGCAGAC<br>GTGCTGGTGTCGGATAAA	Z1657-Z1658	94°C, 30 s	54°C, 60 s	72°C, 60 s	939	83086-83103<br>84024-84007
118	Z1658-1<br>Z1660-2	GCCTTTCCGCTGCTGATG<br>CGACGATTGCTGGGAAGG	Z1658-Z1660	94°C, 30 s	57°C, 60 s	72°C, 60 s	578	83859-83876<br>84436-84419

PCR No.	Primer[a]	Sequence (5′ - 3′)	Target	PCR conditions[b]			PCR product (bp)	Position of the primer[c]
				Denaturing	Annealing	Extension		
119	Z1660-1<br>Z1662-2	ATGATGTCGCTGTTGCTG<br>TAGCCGTTATCGCAGACC	Z1660-Z1662	94°C, 30 s	55°C, 60 s	72°C, 60 s	779	85124-85141<br>85902-85885
120	Z1662-1<br>Z1663-2	GCTACTCCCTTCGTGTTG<br>CGATACAGGGCAACAGTG	Z1662-Z1663	94°C, 30 s	54°C, 60 s	72°C, 60 s	512	85899-85916<br>86410-86393
121	Z1664-1<br>Z1664-2	CTGGCTGAAATCTCGGTGCTG<br>GATCACCCCTCGCTCAAACAC	Z1226, Z1664[f]	94°C, 30 s	57°C, 60 s	72°C, 60 s	327	86689-86709<br>87015-86995
122	LG-1<br>SerWX-2	AAATCAGCGAAGCGAACA<br>TGTCCGAGTGGCTGAAGG	Z1226/Z1664 - *serW/serX*	94°C, 30 s	54°C, 60 s	72°C, 60 s	694	86936-86953<br>25-8[g]
123	LG43<br>InfA-r	GTAAACCAATCCCCGACAACC<br>CCTGACGGGCGACAAAGTGAC	Z1226-*infA* (right junction of OI 43 and the core genome)	94°C, 30 s	55°C, 60 s	72°C, 60 s	781	87188-87208<br>79-59[h]
124	OI 48 CI-F<br>serX-R	TTTGCCAGTATGCTCAAAGA<br>GGTATCGAACGTTGGGTGAT	Z1664 downstr-*ycdW* (right junction of OI 48 and the core genome)	95°C, 45 s	58°C, 45 s	72°C, 90 s	505	87420-87401<br>75-56[i]
125	SerW-3<br>SerW-4	CGCTGGATAAAGAGAAAATG<br>GCAAAAACTACATCCGCATCC	*serW* intact	94°C, 30 s	54°C, 60 s	72°C, 90 s	859	2201-2221[d]<br>98-78[h]
126	serX-F<br>serX-R	TGTCGATTCCTCTGGCATAA<br>GGTATCGAACGTTGGGTGAT	*serX* intact	95°C, 45 s	60°C, 45 s	72°C, 90 s	624	913-932[e]<br>75-56[i]

PCR No.	Primer[a]	Sequence (5´ - 3´)	Target	PCR conditions[b]			PCR product (bp)	Position of the primer[c]
				Denaturing	Annealing	Extension		
127	Z1565F<br>Z1638R	TGAAAGTCTGGCGATGAGTG<br>GTACTACCGTCAGCCGGAAA	internal deletion of OI 48 in strain 134S	95°C, 45 s	60°C, 30 s	68°C, 240 s	2,017	6257-6276<br>62046-62027
128	Z1567F<br>Z1648R	ATGCTGGCGGGATATTACTG<br>GGATTTTCCAGCGTCATGTT	internal deletion of OI 48 in strains 81S/154S	95°C, 45 s	60°C, 30 s	68°C, 240 s	4,825/<br>2,379	7831-7850<br>71571-71552
129	134S-F2<br>134S-R2	CAGAGTTGTACCGGTTATTGAGG<br>CCATTTCGTAATTGGGATTAGC	walking primer for sequencing of PCR product 127	-<br>-	59°C<br>59°C	-<br>-	-<br>-	n.a.[j]<br>n.a.
130	81S-F2<br>81S-F3<br>81S-F4<br>81S-R2<br>81S-R3<br>81S-R4<br>154S-R2	ACGTTAGTGTGGCCAGTTCC<br>ATGATGTCGCTGTTGCTGAC<br>GCCCTAATTATCCTCCCGAAT<br>ACCGGCCAGTCTGCTATG<br>TGCGGTCATCACGAACATAC<br>ATACGGCGGGAGACTTTTTC<br>GTTCCATTGCCCCAAAGATT	walking primer for sequencing of PCR products 128	-<br>-<br>-<br>-<br>-<br>-<br>-	59°C<br>59°C<br>59°C<br>59°C<br>59°C<br>59°C<br>59°C	-<br>-<br>-<br>-<br>-<br>-<br>-	-<br>-<br>-<br>-<br>-<br>-<br>-	n.a.<br>n.a.<br>n.a.<br>n.a.<br>n.a.<br>n.a.<br>n.a.
131	RecAF<br>RecAR	CCGGTAAAACCACGCTGAC<br>CCTGACCGATCTTCTCACCT	RecA	95°C, 30 s	54°C, 45 s	72°C, 60 s	693	212-230[k]<br>904-885[k]
132	TerE1<br>TerE2	TAAAAGGCGGCAACGTATCTCTGA<br>CCGTCCCGTTGTCGTTGTTGTAA	TerE	95°C, 30 s	58°C, 45 s	72°C, 60 s	399	46020-46043<br>46418-46396

PCR No.	Primer[a]	Sequence (5´ - 3´)	Target	PCR conditions[b]			PCR product (bp)	Position of the primer[c]
				Denaturing	Annealing	Extension		
133	Z1567F	ATGCTGGCGGGATATTACTG	5’ junction of internal deletion in strain 81L	94°C, 30 s	53°C, 30 s	68°C, 5 m	3,464	7831-7850
	Z1568-4	ATCGCAGAAAATGAAACC						9981-9964
						-		
	81S-F2	ACGTTAGTGTGGCCAGTTCC	walking primer for sequencing	-	59°C	-	-	n.a.
	81S-F3	ATGATGTCGCTGTTGCTGAC		-	59°C	-	-	n.a.
	Z1568-R1	ACCTGAGGTAATTGCGCTTT		-	59°C	-	-	9360-9341
	Z1568-R2	CCAGCGCAGCAGGTACAC		-	59°C		-	8846-8829
134	Z1637F	CGCTGATTGACTTTCGGTAAT	3’ junction of internal deletion in strain 81L	94°C, 30 s	61°C, 30 s	68°C, 10 m	~6,200	60841-60861
	Z1648R	GGATTTTCCAGCGTCATGTT						71571-71552
	Z1637/38F	ATTCTGGTACCGGTTGATGC	walking primer for sequencing[1]	-	59°C	-	-	61342-61361
	81S-R2	ACCGGCCAGTCTGCTATG		-	59°C	-	-	n.a.
	81S-R3	TGCGGTCATCACGAACATAC		-	59°C	-	-	n.a.
	81S-R4	ATACGGCGGGAGACTTTTTC		-	59°C	-	-	n.a.
	81S-R5	CGGAACGTTACCTCACAGC		-	59°C	-	-	n.a.

PCR No.	Primer[a]	Sequence (5´ - 3´)	Target	PCR conditions[b]			PCR product (bp)	Position of the primer[c]
				Denaturing	Annealing	Extension		
135	Z1565F	TGAAAGTCTGGCGATGAGTG	5' junction of internal deletion in strain 134L	95°C, 30 s	45°C, 30 s	68°C, 5 m	3197	6257-6276
	Z1567-2	TACGCCTCAGAATAATAC						8140-8123
	134S-F2	CAGAGTTGTACCGGTTATTGAGG	walking primer for sequencing	-	59°C	-	-	n.a.
	134L-F1	GTGCTGCTGGTTACCTCGTT		-	59°C	-	-	n.a.
	ECs5244-2	CCAGCCTTCCCAGCAATCGTC		-	59°C	-	-	n.a.
	134L-R1	CTGGAAAGTCAGGGCGAATA		-	59°C	-	-	n.a.
	ECs5244-1	TCAGCAACAGCGACATCATCC		-	59°C	-	-	n.a.
	134S-R2	CCATTTCGTAATTGGGATTAGC		-	59°C	-	-	n.a.
136	Z1636-1	GTTTTCTGGGCTTGTCTC	3' junction of internal deletion in strain 134L	95°C, 30 s	53°C, 30 s	68°C, 5 m	1,446	60601-60618
	Z1638R	GGATTTTCCAGCGTCATGTT						31082-31063
	Z1638-2	GGCAGAAGCAGAAAAAGC	walking primer for sequencing	-	52°C	-	-	61529-61512

PCR No.	Primer[a]	Sequence (5´ - 3´)	Target	PCR conditions[b]			PCR product (bp)	Position of the primer[c]
				Denaturing	Annealing	Extension		
137	Z1567F	ATGCTGGCGGGATATTACTG	5’ junction of internal deletion in strain 154L	94°C, 30 s	61°C, 30 s	68°C, 10 m	3,213	7831-7850
	Z1574R	CGACTGCTGCACCTTCATAA						15403-15384
	ECs5244-2	CCAGCCTTCCCAGCAATCGTC	walking primer for sequencing	-	59°C	-	-	n.a.
	81S-F3	ATGATGTCGCTGTTGCTGAC		-	59°C	-	-	n.a.
	Z1573R	ACAGGGCTTTACCTGTCAGC		-	59°C	-	-	14772-14753
	Z1573-2	CCGCTCATCACTTTGTTT		-	50°C	-	-	14384-14367
	ECs5243-1	GCGATTCGTATGGTTCTGGA		-	59°C	-	-	n.a.
	ECs5244-1	TCAGCAACAGCGACATCATCC		-	59°C	-	-	n.a

PCR No.	Primer[a]	Sequence (5´ - 3´)	Target	PCR conditions[b]			PCR product (bp)	Position of the primer[c]
				Denaturing	Annealing	Extension		
138	Z1640F	CTGCTGGATTTACGGGACAT	3’ junction of internal deletion in strain 154L	94°C, 30 s	60°C, 30 s	68°C, 10 m	6,424	63370-63389
	Z1648R	GGATTTTCCAGCGTCATGTT						71571-71552
	Z1640-3	GTTTGTGGCTGGTGTCTG	walking primer for sequencing	-	54°C	-	-	63786-63803
	Z1641-1	GAAATGACGACAGAGAGG		-	50°C	-	-	64220-64237
	Z1642-3	GAAAAGATGGACAAACAG		-	49°C	-	-	64863-64880
	Z1642F	GAGAAGGCTGGCGCTATTTA		-	59°C	-	-	65291-65310
	Z1642F2	TCAGGAACATGGTGCCAGTA		-	59°C	-	-	65800-65819
	ECs5244-2	CCAGCCTTCCCAGCAATCGTC		-	59°C	-	-	n.a.
	81S-F3	ATGATGTCGCTGTTGCTGAC		-	59°C	-	-	n.a.
	ECs5243-1	GCGATTCGTATGGTTCTGGA		-	59°C	-	-	n.a.
	ECs5244-1	TCAGCAACAGCGACATCATCC		-	59°C	-	-	n.a.
	Z1647R	AAACCGTGCAGAAGTGGAAC		-	59°C	-	-	71046-71027
	Z1642/43R	CACACCTGGAAAATCCATGC		-	59°C	-	-	67171-67152
	Z1642R	AATGGAGTCTTCCCGACCTT		-	59°C	-	-	66661-66642

a. Primers were derived from the sequence of O island (OI) 48 of *E. coli* O157:H7 strain EDL933 (GenBank accession number AE005174; genomic position 1454242 to 1541789), which is identical in its nucleotide sequence to OI 43 of EDL933, and from the genes flanking OI 48 (*ycdU*, *serX*, *ycdW*) and OI 43 (*clpA*, *serW*, *infA*) in the genome of strain EDL933.

b. All PCRs included 30 cycles as indicated, preceded by denaturation (94°C, 5 min) and followed by a final extension (72°C, 5 min).

c. Positions of PCR primers within the sequence of OI 48 of *E. coli* O157:H7 strain EDL933 are indicated unless specified otherwise; position of the first nucleotide of OI 48 (1454242) is considered as position 1.

d. Position of the primer within *clpA* (Z1119), which is located directly upstream of the integrase gene of OI 43 in strain EDL933 (Fig. S1A).

e. Position of the primer within *ycdU* (Z1558), which is located directly upstream of the integrase gene of OI 48 in strain EDL933 (Fig. S1A).

f. Last genes of OI 43 and OI 48, respectively.

g. Positions of the primer within *serW* and *serX* genes (which are 100% identical).

h. Position of the primer within *infA* (Z1228), which is located downstream of OI 43 (Fig. S1A).

i. Position of the primer within *ycdW* (Z1666) which is located downstream of OI 48 (Fig. S1A).

j. n.a., not applicable

k. Position of the primer within *recA*(Z4002).

l. Full sequencing of the PCR product was not possible due to the presence of an additional IS*629* besides Z1638/Z1339 in the PCR fragment. However, the existence of two IS elements has been verified by restriction digestion with *Hind*III, a single cutting enzyme in IS*629*, which resulted in three restriction fragments of the expected size (data not shown). According to sequencing data additional *Hind*III restriction sites in flanking regions were excluded.

Table S2. PCR primers for analysis of the core genome deletions in strain 95S.

Primer	Sequence (5´- 3´)	Target	Annealing temperature	PCR product (bp)
ycdU-1<br>ycdU-2	GTGGGATTCTGGCCCTGGTGC<br>GCGTACCGCCTGGAGAAGCG	Z1558<br>(*ycdU*)	59°C	538
Z1549-1<br>Z1549-2	GCACTGAGCCCATCTATC<br>ATTCGGCACATAGTCCAG	Z1549	55°C	554
Z1539-1<br>Z1539-2	AGCCATCACTATCACAAGCAA<br>CCTTCAGGGATTAAGTGCAAA	Z1539	58°C	494
Z1528-1<br>Z1528-2	TGATTGCTAACGGAGCATTG<br>GCAGCGCGGATAAAAACTAC	Z1528	59°C	712
Z1522-1<br>Z1522-2	GCATTTTCAACCTCATCC<br>TCCACCTTCCCTATTTCC	Z1522	54°C	674
Z1513-1<br>Z1513-2	CCAGGCGAATCATAAGAC<br>TCGCTGAACCGCATTATC	Z1513	55°C	607
Z1505-1<br>Z1505-2	AATCAGCCCGAACACAAC<br>CTGGATGGCGGTAATGAC	Z1505	55°C	574
Z1495-1<br>Z1495-2	GGCAACTGTTACGCACTC<br>CGAATGCCTCACGAATAC	Z1495	55°C	1224
Z1486-1<br>Z1486-2	CCGAATGATGATGAAACC<br>ACATCCTGCTTACCATCC	Z1486	54°C	791
Z1477-1<br>Z1477-2	CGTGAAGCCTGGTTTGTC<br>GCCCCTGAATAACCTCTG	Z1477	55°C	784
LP43<br>LP44	ATCCTATTCCCGGGAGTTTACG<br>GCGTCATCGTATACACAGGAGC	Z1464	57°C	584
wrbA1<br>wrbA2	ATGGCTAAAGTTCTGGTG<br>CTCCTGTTGAAGATTAGC	Z1423	53°C	600
Z1409-1<br>Z1409-2	TGCGTAACCAATCACCGTAA<br>CTGAATTAATCGGCGCTCTC	Z1409	59°C	605
Z1408-1<br>Z1408-2	GGCTCCCTTGTGGAGCCTTTTT<br>CGCCAGTAAGGTGTTGGTTCCCG	Z1408	57°C	155

Primer	Sequence (5´- 3´)	Target	Annealing temperature	PCR product (bp)
Z1406-1<br>Z1406-2	TCCTGATGATGGCAGCAAAGACG<br>GGGCCACGTTGACCCTGCTC	Z1406	57°C	120
Z1405-1<br>Z1405-2	CGCTGTTGGTCCTCGCAGGC<br>ACCTTTGATCGCAAAAGCGGCA	Z1405	58°C	162
Z1404-1<br>Z1404-2	GCGCGTAGTGGACGTGGTGG<br>GACGCCAGCTGCGTTTGCTG	Z1404	59°C	259
Z1403-1<br>Z1403-2	ACACAGCACATCGCGACCCG<br>TCAGCAGATCCAGGCGTTGCC	Z1403	59°C	364
Z1402-1<br>Z1402-2	CACAGGGCAACAGGGGCGAC<br>TTCCGTTGGCCCCGTGGAGA	Z1402	60°C	504
Z1401-1<br>Z1401-2	TTGCGCAGGCTGGCTACGTC<br>TGGCGCTGTTCCCATGGCTG	Z1401	59°C	946
Z1400-1<br>Z1400-2	AGCGTTACCCAGCGCTTCCG<br>CGCAGGCTAACCCGGAGCTG	Z1400	59°C	647
Z1399-1<br>Z1399-2	GACCTCGGGAGCGATTGCGG<br>CATTGGCGAGCGCTTGCTGC	Z1399	59°C	241
Z1398-1<br>Z1398-2	GCAGGCTGGTGCCGACAGAG<br>GAAGTGCGCAGCGAGCTGGA	Z1398	60°C	1174
Z1397-1<br>Z1397-2	TCGTCATGGTGTGCGTGCCC<br>ATCCTTCCCCACCCCGGCTC	Z1397	59°C	666
Z1396-1<br>Z1396-2	TAGCGTTGGTGCGCACTGGG<br>CAGCGTGATGCCTGCCGTGA	Z1396	59°C	775
Z1395-1<br>Z1395-2	CCTGCTGGTGACGTGGCTGG<br>ACGGCAAACTCCAGAGCGCC	Z1395	59°C	917
Z1394-1<br>Z1394-2	ACAGCCCGCAGGTGATTG<br>TCAATGGGCGGCGGTAAC	Z1349	58°C	352
Z1376-1<br>Z1376-2	TCCACGGGGCTGAAAATC<br>GGAGAGCGTTTTCGTCAC	Z1376	56°C	375
Z1349-1<br>Z1349-2	GTGAGGGCGTTTATTTTG<br>GCATTCAGTCCCAGTGTC	Z1349	56°C	844

Primer	Sequence (5´- 3´)	Target	Annealing temperature	PCR product (bp)
Z1329-1<br>Z1329-3	GCAGTTCCGGTAATCCTGTG<br>AATCCAGGAAATTGCAGCAC	Z1329	60°C	331
Z1309-1<br>Z1309-2	ACCGATCATTGTTTGGCAGT<br>GAAACTCCTGTTTCGGCGTA	Z1309	60°C	543
Z1297-1<br>Z1297-2	GGCTGACGGTGGTGAAAATAC<br>GTTTTGCTGATTTCGCCACAC	Z1297	56°C	405
Z1280-1<br>Z1280-2	AAATCCTGACGCTGCCTTCTG<br>CAGGTCGGTAAGCATTTCCAC	Z1280	58°C	602
Z1270-1<br>Z1270-2	ATCCCTCGTTCCGTCTTGTCG<br>TGGCTGATTCTCTTCGGTTTC	Z1270	57°C	438
Z1256-1<br>Z1256-2	TGGCACTAGCGGCATTACATC<br>CCGAGGAGGCGTCAAGGAAAA	Z1256	57°C	327
Z1243-1<br>Z1243-2	CAGGGCGAGCAATGTAAGGTG<br>ATGATGCTGCCGTTTTGCTTG	Z1243	55°C	225
Z1232-1<br>Z1232-2	TTTGATGTAGCCGTTTTCCAG<br>CCGTGGAGTTTCTGCTTGTGC	Z1232	56°C	406
clpA-1<br>clpA-2	GAATCAGCTTGCGCGCGTGG<br>TTCGTCCAGCAGCAGCACCG	Z1119<br>(*clpA*)	59°C	1168
Z1118-1<br>Z1118-2	TTCGCGACGCGCTAAAACCG<br>TGGTTTCTGCAACCTCGGCGG	Z1118	59°C	198
Z1117-1<br>Z1117-2	TGCCTTTTGGCCCCTGGTGG<br>CATCTGCCCTGAAGGCGGCG	Z1117	59°C	125
Z1116-1<br>Z1116-2	TGCTTCGCGCCCAGGAGTTG<br>ACCTGCGCACGACCGTTCAG	Z1116	59°C	838
Z1115-1<br>Z1115-2	TGCTGGCGACCGGAAAGCTG<br>CCGGATCGCCTAACGCCGAC	Z1115	59°C	804
Z1112-1<br>Z1112-2	CGCCGACGGTATTCAGCCCG<br>ATCGCCCTTACCTTGCGGCG	Z1112	59°C	649
Z1110-1<br>Z1110-2	CTGGCGTCTGGGGCCAAGTG<br>CAGCCACAGCACGCGTGAGA	Z1110	59°C	595

Primer	Sequence (5´- 3´)	Target	Annealing temperature	PCR product (bp)
Z1109-1<br>Z1109-2	CAGCCGCCCTGGAAGATGGC<br>ATTGCCCAGGTTGTCGGCGG	Z1109	59°C	344
Z1108-1<br>Z1108-2	CGCGCTCCCGATTACCGGAC<br>CGCTTCGCCAACAAGCTGCG	Z1108	59°C	617
Z1107-1<br>Z1107-2	AGCATTTCGCCGTGGGTGTAG<br>GCAGGGGATGTGTGGTAAAAC	Z1107	57°C	762
Z1097-1<br>Z1097-2	CGCGGAAAATATTCCAACAG<br>TCATCAGCAAATCCAAGCAC	Z1097	59°C	573
Z1087-1<br>Z1087-2	TGGTTTGCTGGTGGGTATTGG<br>CGGCATCTTTGGTAACTTTGG	Z1087	55°C	500
Z1074-1<br>Z1074-2	TCGGCATCTCAATCTGGCTAC<br>CATTGGCGTTTTAGTCGTATC	Z1074	56°C	908
Z1063-1<br>Z1063-2	ATTCCGAAGTTGGCGATGATG<br>GCCGTAGTTTTTGCCTTTTTG	Z1063	56°C	443
Z1054-1<br>Z1054-2	TTTGCTTTCCCCATTCCTTAC<br>CTCTTTTTGCCCTTTACCTTC	Z1054	56°C	480
Z1045-1<br>Z1045-2	TTACCCACCGAGCCAATGACC<br>TCCACTCAGCCTAACCTCAGC	Z1045	58°C	998
Z1035-1<br>Z1035-2	TAGCAGGAACCACAGACCAAG<br>TGGCGTCTATTCAAGGTGGAG	Z1035	57°C	347
Z1026-1<br>Z1026-2	ACCCATAATCGCCGTCATCAG<br>GGATACTACCCGCACGATGAC	Z1026	58°C	826
Z1017-1<br>Z1017-2	CCACGGTAATAAATCGCAAGG<br>TGATTTCGCACCACCTTCTCC	Z1017	58°C	436

All primers were designed in this study except for LP43 and LP44 (Friedrich *et al.,* 2002) and wrbA1 and wrbA2 (Bielaszewska *et al*., 2006b).

Table S3. Quantitative real time RT-PCR assays to determine transcription of *iha* and non-*iha* adhesins in L and S strains.

Primer	Sequence (5´ - 3´)	Target	Annealing temperature	PCR product (bp)	Reference
GapA_for<br>GapA_rev	GTTGTCGCTGAAGCAACTGG<br>AGCGTTGGAAACGATGTCCT	*gapA*	53°C - 60°C	171	Blumer *et al*., 2005
K14924<br>K14925	TTACCAGCGATACCAAGAGC<br>CAACATGACCGATGACAAGG	*eae*	59°C	126	Chen *et al*., 2007
CMD306<br>CMD307	GGCTGAATCTGCAGGAAAGCAACA<br>TGCAGGCTGACAGAATCATCCACA	*iha*	60°C	95	Léveillé *et al.,* 2006
ehaA-F<br>ehaA-R1	AGCGTTAGGGGCACAGGAAG<br>CCTCGCTGCCACTGACAATA	*ehaA*	54°C	161	This study[a]
LPFA1-CF<br>LPFA1-CR1	GGTTGGTGACAAATCCCCG<br>CGTCTGGCCTTTACTCAGA	*lpfA*1<br>(O157/OI-141)	53°C	244	Torres *et al.,* 2009
LPFA2-F1<br>LPFA2-CR	GCAGGTCACCTACAGGCGGC<br>GCTAATACCAGCGGCAGCATCGT	*lpfA*2<br>(O157/OI-154)	57°C	306	This study[b]<br>Torres *et al.,* 2009

a. The primers were derived from a 851-bp fragment of *ehaA* passenger domain sequenced in our laboratory, which was 100% identical in all L and S strains and also to the respective sequences in EHEC O157:H7 strains EDL933 and Sakai (data not shown).
b. The primer was derived from the sequence of *lpfA2* of strain EDL933 (GenBank accession no. AE005174).

Table S4. Plasmids and *E. coli* constructs used in this study.

Plasmid or construct	Description	Source or reference
**Plasmids**		
pWKS30	*ori* pSC101, *bla*	Wang and Kushner, 1991
pWKS30*iha*	pWKS30 with a 2,916-bp fragment containing *iha* from EHEC O157:H7 Sakai (RIMD 0509952)	This study
pKD3	*bla* FRT *cat ori*Rγ	Datsenko and Wanner, 2000
pKD46	*bla araC*-P*araB*-γ-β-exo *ori*R101 *repA*101 (ts)	Datsenko and Wanner, 2000
pKD4	*kan* FRT *cat ori*Rγ	Datsenko and Wanner, 2000
pKD4*iha*	pKD4 with a 2,916-bp fragment (*BstB*I/*Hind*III) containing *iha* from EHEC O157:H7 Sakai (RIMD 0509952)	This study
pCP20	*bla cat flp* (ts)	Cherepanov and Wackernagel, 1995
pGEM-T easy	*bla lacZ*α	Promega
pTerE	pGEM-T Easy containing a 399-bp internal *terE* fragment	This study
**Constructs**		
154LΔ*iha*	154LΔ*iha*	This study
154LΔ*iha*/pWKS30*iha*	154LΔ*iha* containing pWKS30*iha*, Ap^R	This study
154LΔ*iha*/pWKS30	154LΔ*iha* containing pWKS30, Ap^R	This study
154S *glmS*::*iha*	154S chromosomally complemented (downstream of *glmS*) with *iha* from EHEC O157:H7 Sakai (RIMD 0509952), Km^R	This study
SakaiΔ*iha*	EHEC O157:H7 Sakai (RIMD 0509952)Δ*iha*	This study
SakaiΔ*iha glmS*::*iha*	EHEC O157:H7 SakaiΔ*iha* chromosomally complemented (downstream of *glmS*) with *iha* from EHEC O157:H7 Sakai (RIMD 0509952), Km^R	This study

Table S5. Primers used for construction of *iha* deletion and complementation mutants.

Primer[a]	Sequence (5′ - 3′)
del_iha_for	GTATTTGTGTATTGTCTTGCCGGTTAACATGATCGGAGATTAGTAATATGGTGTAGGCTGGAGCTGCTT
del_iha_rev	TGCAGTGGCAACGTATTCTACCGTCAGTGATAGCGTTTTGTTATTATCACATATGAATATCCTCCTTAGTTCCTA
iha_for2	GGTTTCGAACAATGTGGGCTGACATGA
iha_rev	CGGTAGCTGCAAAACTTAACG
iha-pKD4_for	CCTTTTTGCGTGGCCAGTGCCAAGCTTGCATGCAGATTGCAGCATTACACAACAATGTGGGCTGACATGA
iha-pKD4_rev	AAAGTATAGGAACTTCGAAGCAGCTCCAGCCTACACAATCGCTCAAGACCGGTAGCTGCAAAACTTAACG
iha-int_for	AACTCCATTAATTTGATATTATTCCTGTGTGTTATCCATTTATTAAATTTAACAATGTGGGCTGACATGA
iha-int_rev	ACTTACCAGCATTAATAATCTAACCTCATTCCATATAATTAACTTCAGCTACGGCTGACATGGGAATTAG

a. All primers were designed in this study.
